# Limited dissemination of the wastewater treatment plant core resistome

**DOI:** 10.1038/ncomms9452

**Published:** 2015-09-30

**Authors:** Christian Munck, Mads Albertsen, Amar Telke, Mostafa Ellabaan, Per Halkjær Nielsen, Morten O. A. Sommer

**Affiliations:** 1 Novo Nordisk Foundation Center for Biosustainability, Technical University of Denmark, Kogle Alle 6, DK-2970 Hørsholm, Denmark; 2 Center for Microbial Communities, Department of Chemistry and Bioscience, Aalborg University, 9220 Aalborg, Denmark; 3 Department of Systems Biology, Technical University of Denmark, DK-2800 Lyngby, Denmark

## Abstract

Horizontal gene transfer is a major contributor to the evolution of bacterial genomes and can facilitate the dissemination of antibiotic resistance genes between environmental reservoirs and potential pathogens. Wastewater treatment plants (WWTPs) are believed to play a central role in the dissemination of antibiotic resistance genes. However, the contribution of the dominant members of the WWTP resistome to resistance in human pathogens remains poorly understood. Here we use a combination of metagenomic functional selections and comprehensive metagenomic sequencing to uncover the dominant genes of the WWTP resistome. We find that this core resistome is unique to the WWTP environment, with <10% of the resistance genes found outside the WWTP environment. Our data highlight that, despite an abundance of functional resistance genes within WWTPs, only few genes are found in other environments, suggesting that the overall dissemination of the WWTP resistome is comparable to that of the soil resistome.

## Abstract (web summary)

Wastewater treatment plants (WWTPs) are thought to play a central role in the dissemination of antibiotic resistance genes. Here, the authors identify novel antibiotic resistance genes in WWTPs and show that only a few of the most abundant resistance genes are found outside the WWTP environment.

## Graphical abstract

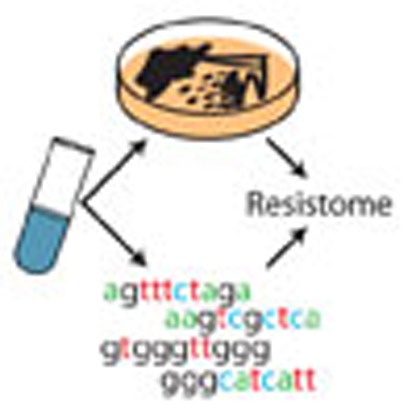

Investigations of environmental sources of antibiotic resistance genes have shown that many clinically relevant resistance genes such as the cephalosporin resistance gene family *ctx-m*, the quinolone resistance gene family *qnr* and the vancomycin resistance gene family *van* have close homologues on the chromosomes of environmental non-pathogenic bacterial species^1^,^2^,^3^,^4^,^5^. These findings highlight that resistance genes can spread from environmental reservoirs to human pathogens. This has led to a growing interest in uncovering which environmental microbial communities participate most in antibiotic resistance gene exchange with the aim of establishing the most impactful policies that limit the dissemination of antibiotic resistance^3^,^4^,^6^,^7^,^8^.

To assess the resistance gene load of a given environmental microbial community, researchers have used targeted PCR or metagenomic read mapping to detect the presence of well-characterized resistance genes^7^,^9^. These studies have shown that representatives of clinically relevant resistance genes can be found in virtually all environments. Although such studies are important and relevant, they rarely consider the abundance of the targeted resistance genes relative to the complete resistome of the studied environment. This is largely due to our incomplete annotation and knowledge of antibiotic resistance genes^10^. Indeed, less biased methods such as metagenomic functional selections have revealed that <1% of the resistance genes found in soil have high identity (>99% nucleotide identity) hits in NCBI databases^8^,^11^. Accordingly, the majority of the soil resistome has not been found outside the soil environment. On the other hand similar approaches have been used to show that a subset of the human gut microbiome may exchange resistance genes with human pathogens^12^. So far, metagenomic functional selections have largely been applied to study the resistomes of soil and gut microbial communities; yet, other communities are believed to contribute substantially to the dissemination of resistance genes. Hence, to make informed policy decisions addressing the challenges associated with antibiotic resistance, the abundance and dissemination of antibiotic resistance genes in these communities must be characterized.

Wastewater treatment plants (WWTPs) are generally considered to be important hubs for horizontal gene transfer of antibiotic resistance genes^13^,^14^,^15^. In these facilities, wastewater from diverse sources such as hospitals, households and animal production farms is mixed, often containing both pathogenic bacteria and traces of antibiotics^16^,^17^,^18^. Accordingly, the dense microbial communities of WWTPs should provide ideal conditions for horizontal exchange of antibiotic resistance genes between a wide range of bacterial species, including human pathogens.

Several PCR-based studies have shown that clinically relevant resistance genes, including *tem1*, *ampC*, *ndm-1*, *vanA* and *ermB*, can be found in WWTPs^19^,^20^,^21^,^22^. Similarly, metagenome sequencing studies characterizing either complete or closed circular metagenomes (plasmids and some phages) have used sequence read mapping to resistance gene databases, to identify several different resistance genes in WWTPs^9^,^23^,^24^,^25^,^26^. Furthermore, traditional cultivation-based studies have also shown that vancomycin-resistant enterococci, methicillin-resistant staphylococci and cephalosporin-resistant Enterobacteriaceae can be isolated from WWTPs^20^,^27^. However, owing to the specificity of PCR-based screening and database mapping, these approaches can only identify known resistance genes and their close homologues. Consequently, such methods cannot uncover the full spectrum of functional resistance genes within an environment. Likewise, cultivation-based approaches will only recover a small proportion of bacterial species, with estimates indicating that 85%–99% of the bacteria from WWTPs are not readily cultured using current protocols^28^. Here we combine functional selection and metagenomic sequencing to characterize and trace the most abundant antibiotic resistance genes in the WWTP environment. We find that only a small subset of the core WWTP resistome is found outside the WWTP environment.

## Results

### Metagenomic functional selection

Using metagenomic functional selection, we identified the dominant members of the resistome in a large modern WWTP, Aalborg West (AAW), sampled in the third quarter of 2012. This facility is located in Denmark and receives both hospital and household wastewater, and has been in operation since 1979 (Supplementary Table 1 and Methods). A total of 800 Mbp of DNA was cloned in an *Escherichia coli* expression library (Supplementary Methods) and screened on 15 different antibiotics representing 8 chemical classes: β-lactam, aminoglycoside, macrolide, tetracycline, phenicol, rifamycin, sulphonamide and dihydrofolate reductase inhibitor (Table 1). In total 8,540 resistant clones were identified with an average of 534 colonies per antibiotic ranging from 30 clones for chloramphenicol to more than 2,000 clones for trimethoprim (Table 1). Of the 8,540 identified clones, the inserts of 749 clones were selected proportionally among the classes of antibiotics and sequenced, resulting in the identification and annotation of 79 unique functionally validated resistance genes (Supplementary Table 2). Rarefaction curve analysis showed that we sampled the majority of the resistance genes in the library (Supplementary Fig. 1). Accordingly, these genes are expected to represent the most abundant genes in the WWTP resistome and we refer to them as the core WWTP resistome.

### WWTP community stability

**Figure 1:**
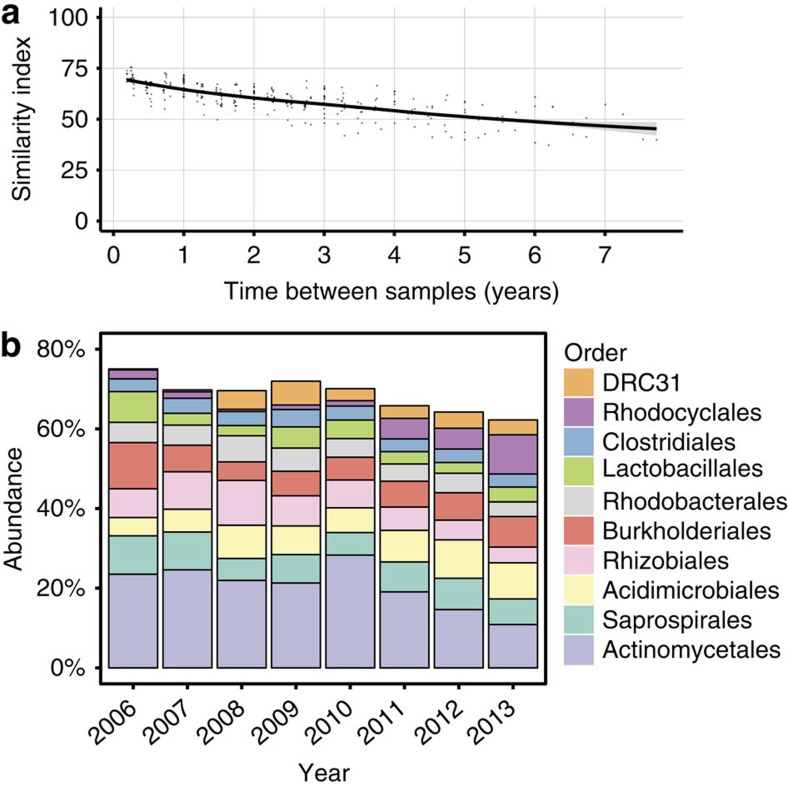
Phylogenetic analysis of WWTP AAW. (**a**) The pairwise similarity of the microbial community in samples collected from the WWTP AAW over a 7-year period (*n*=24). Similarity is calculated as (1−Bray–Curtis dissimilarity index) × 100 for each pairwise comparison of 16S rRNA amplicons and plotted against the time (years) between the samples. (**b**) Per cent abundance of the ten most abundant bacterial orders in WWTP AAW over a 7-year period. On average, 68% (s.d.=4%, *n*=24) of the 16S rRNA gene sequences belong to these orders.

Considering the constant flow of material through the AAW WWTP, up to 50,000 m^3^ per day, it could be expected that the microbial community varied greatly over time. However, using a modified version of the Bray–Curtis dissimilarity index ((1−Bray–Curtis dissimilarity index ) × 100), we calculated the similarity between all pairwise combinations of samples collected from AAW over a 7-year period (*n*=24) (Fig. 1a)^29^. Sample similarity was indexed from 0 to 100, with 0 indicating completely disjoint sample and 100 indicating identical samples. The comparison revealed that the bacterial communities were remarkably stable (Fig. 1a). Notably, samples collected 7 years apart had an average similarity index of 48 (Fig. 1a) and samples collected <1 year apart had an average similarity index of 68, highlighting that the WWTP microbial community is highly stable and suggesting that variation in wastewater inflow has a limited impact on the core bacterial communities.

Comparison of the relative abundances of the ten most abundant bacterial orders in the WWTP community across the 7 years of sampling showed that the WWTP community is highly stable and unique to this environment (Fig. 1b). As recently reported, the WWTP environment is dominated by the phyla Actinobacteria (Actinomycetales and Acidimicrobiales), Bacteriodetes (Saprospirales) and Proteobacteria (Rhizobiales, Burkholderiales and Rhodobacterales), highlighting that WWTPs contain distinct microbial communities^30^. Still, owing to horizontal gene transfer, a stable microbial community at the 16S ribosomal RNA sequence level does not necessarily imply a stable resistome.

### WWTP core resistome stability

**Figure 2:**
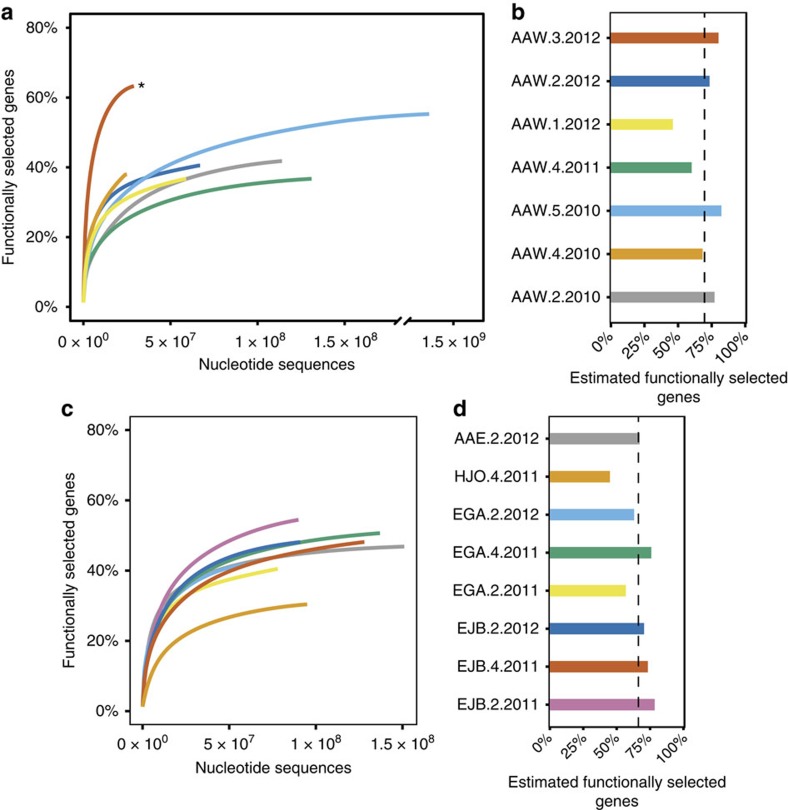
Tracing the functionally selected resistome. (**a**) Rarefaction curves showing the percentage of the functional selected resistance genes found in the AAW metagenomes sampled over a 2-year period. The curves are based on read mapping (>95% identity) to the functional selected resistance genes (Methods). For each sample, the corresponding sample name is given by the colour code in **b**. The asterisk denotes the sample used in the functional selection. (**b**) Rarefaction curve-based predictions of the expected percentage of functionally selected resistance genes present in each of the AAW WWTP samples. Samples are named according to the collection time in the format: Location.Quarter.Year (quarter 5 refers to December). The dashed line indicates the average expected abundance. Predictions are made using the model described in Chiu *et al*.^64^ with the R package Vegan^62^. (**c**) Rarefaction curve showing the percentage of the functional selected resistance genes found in the non-AAW WWTP metagenomes generated as in **a**. For each sample, the corresponding sample name is given by the colour code in **d**. (**d**) Rarefaction curve-based predictions of the percentage of functionally selected resistance genes present in each of the non-AAW WWTP samples. Sample names are generated as in **b**. The dashed line indicates the average expected abundance. Despite being distinct from the AAW WWTP, the non-AAW WWTPs are estimated to contain the majority of the resistance genes found in AAW.

To investigate the long-term stability of the WWTP core resistome, we sequenced seven metagenomes spanning 2 years from the same WWTP used for the metagenomic functional selections (AAW; Supplementary Table 3). Approximately 230 Gbp of sequence data corresponding to more than 1.5 billion raw reads were sequenced. By mapping the metagenomic sequencing reads to the functional selected resistance genes, we could trace the WWTP core resistome dynamics in the WWTP over the 2-year period (Fig. 2a). Extrapolation of the rarefaction curves predicted that the samples collected over the 2-year sampling period on average contained 70% (s.d.=13%) of the whole resistance gene set (Fig. 2b). This highlights that despite vast amounts of wastewater passing through the treatment plant the resistome remains stable and we consider this set of genes part of the WWTP core resistome.

To evaluate the impact of sequencing depth, we exhaustively sequenced sample AAW.5.2010, which was collected 2 years before the sample used for the functional selection. In this metagenome, consisting of more than 1.1 billion reads, we identified 67% of the functional selected resistance genes and extrapolations of the rarefaction curve estimated that up to 82% genes could be found in this sample (Fig. 2a,b). This further underscores that the core resistome was largely stable during the 2-year sampling period.

### Core resistome hosts

To identify possible hosts carrying the functional selected resistance genes, we assembled contigs from the deeply sequenced metagenome (AAW.5.2010). The relative low coverage of the functionally selected resistance genes challenges their assembly. In addition, the complexity of the WWTP community also challenges the assembly. A total of 43% of all metagenomic reads could be mapped back to the assembled contigs. Seven contigs contained functional selected resistance genes (>95% identity and >90% gene coverage). The contigs were blasted against the genbank nt database, to identify host organisms. In general, the query coverage was low, indicating that the contigs assembled from the metagenomes are located on hitherto uncharacterized genomes, which is largely a result of the limited availability of WWTP-specific bacterial genomes^31^. However, a small contig containing CAR_05 had high identity and query coverage to the *tem-1* β-lactamase gene on cloning vectors (Supplementary Table 4). The challenges of assembling the low coverage genes from the metagenome highlight the power of combining functional selection and metagenomic sequencing, to trace functionality through different metagenomes.

### The WWTP core resistome is stable across multiple facilities

To explore whether the AAW WWTP core resistome was shared with other WWTP facilities, we sequenced additional eight metagenomes from four different Danish full-scale WWTPs: Egaa, Ejby, Hjoerring and Aalborg East. Two of the plants (Egaa and Ejby) were sampled repeatedly over a 1-year period and all plants, except Aalborg East, received household as well as hospital wastewater (Supplementary Table 3). In total, these additional WWTP metagenomes consisted of >124 Gbp corresponding to >800 million reads. Mapping of the metagenomic reads to the AAW core resistome revealed that the majority of the resistance genes were present in the different metagenomic samples (Fig. 2a,c). Extrapolations of the rarefaction curves estimate that on average the non-AAW WWTPs contained 66% (s.d.=11%) of the functionally selected resistance genes from AAW (Fig. 2c,d).

The stability of the WWTP microbial community and core resistome could support the hypothesis that resistance gene exchange within WWTPs is a rare event as was recently reported for soil microbial communities^11^. However, as activated sludge in WWTPs is continuously mixed and exposed to varying levels of antibiotics, one would expect that horizontal gene transfer of resistance genes would be more frequent in WWTPs compared with soil^16^,^17^,^18^. To investigate whether the WWTP core resistomes correlated with the microbial composition, we used procrustes analysis to correlate resistomes and 16S rRNA sequences from each of the 15 WWTP metagenome samples^11^. Our analysis shows that WWTP resistome and phylogeny correlated (*P*<0.001, based on 999 permutations), indicating that the WWTP core resistome is linked to the microbial community (Supplementary Fig. 2). This supports the hypothesis that the WWTP resistome is only exchanged to a limited extent within the WWTP environment.

### Dissemination of the WWTP core resistome

**Figure 3:**
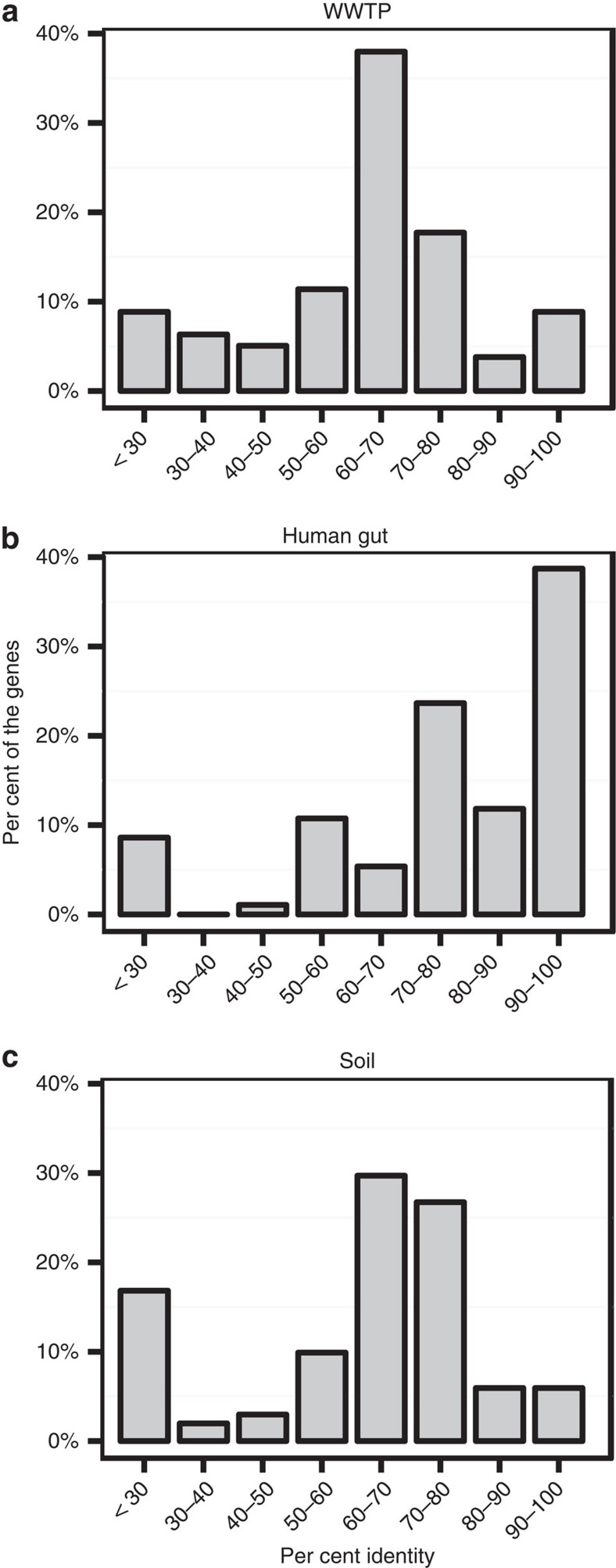
Genbank nucleotide database hit identity. Functionally selected resistance genes from (**a**) WWTP AAW (*n*=79), (**b**) human gut (*n*=94)^12^,^32^ and (**c**) soil (*n*=101)^33^,^34^,^35^,^36^ were blasted against the Genbank nt database and the top hit was aligned to the query. The panels show the nucleotide identity distributions of the pairwise alignment for each environment (Methods).

Recent studies based on metagenomic read mapping showed that the metagenomic sequence data from WWTPs mapped to a distinct set of resistance genes compared with other environments^23^. However, this finding could arise from database biases resulting in an inability to identify shared resistance genes or it could suggest that the WWTP resistome is only disseminated to a limited extent to other environments. To investigate whether the WWTP core resistome is involved in gene exchange networks comprising other environments, as well as human pathogens, we used BLAST to perform a homology search of the WWTP core resistome to genes in the the NCBI nucleotide database. We found that the WWTP resistance genes had an average sequence identity of 62% to the best hit in the database, and that just six genes had an identity >95%, demonstrating that the vast majority of the functional selected resistance genes represent novel sequences not captured by the current antibiotic resistance gene databases (Fig. 3a).

To put this finding into perspective, we re-analysed the available functional selected resistance genes from the human gut microbiome^12^,^32^ and from the soil microbiome^33^,^34^,^35^,^36^ (Fig. 3b,c). We found that functionally selected resistance genes from the human microbiome had an average of 75% identity to genes in the NCBI nucleotide database, whereas functionally selected resistance genes from soil had an average identity of 59%. Notably, 33% of the resistance genes from the human microbiome had >95% identity to genes found in the NCBI nucleotide database, whereas none of the soil genes were above this threshold. Owing to database biases, it is expected that genes from the human microbiome have higher identity to the NCBI nucleotide database. However, considering the large extent to which the human microbiota and human clinical isolates have been sequenced, the finding that only a few number of genes from the WWTP core resistome have an identity >95% to genes in the NCBI nucleotide database indicates that the WWTP core resistome is only disseminated to the human microbiota or human clinical isolates to a limited extent. This probably reflects the fact that the WWTP microbial community is unique to the WWTP environment, and that the majority of the genes in the WWTP core resistome belong to this community.

**Figure 4:**
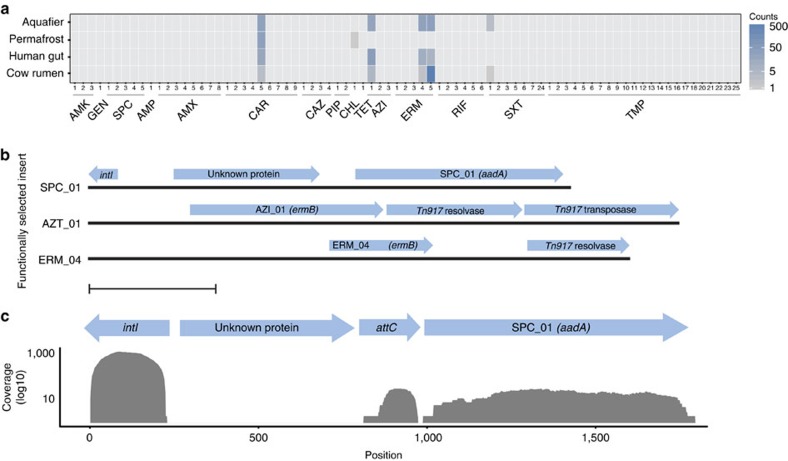
WWTP resistome dissemination. (**a**) Mapping of metagenomic reads from aquifer, permafrost, human gut and cow rumen, to the functionally selected resistance genes. Mapping reads have >95% identity and >95% coverage. The functionally selected resistance genes are grouped by the antibiotic they were selected on as denoted by the three-letter code (AMK (amikacin), AMP (ampicillin), AMX (amoxicillin), AZI (azithromycin), CAM (chloramphenicol), CAR (carbenicillin), CTZ (ceftazidime), ERY (erythromycin), GEN (gentamicin), PIP (piperacillin), RIF (rifampicin), SPC (spectinomycin), SXT (sulfamethoxazole/trimethoprim), TET (tetracycline), TMP (trimethoprim)). (**b**) The flanking regions of three functional selected resistance genes contained elements, indicating that the genes were either located in an integron (SPC_01) or on a transposon (AZT_01 and ERM_04). Blue arrows denote the different genes and their orientation. Scale bar, 500 bp. Genes were annotated using BLAST. (**c**) Read coverage (>95% identity and >95% coverage) of the integrase gene (*intI*) and the attachment site sequence (*attC*) found in the integron carrying SPC_01 in sample AAW.5.2010. The integrase was 50 times more abundant than the SPC_01 resistance gene *aadA*. The blue arrows denoted genes and functional regions within the integron as annotated by BLAST.

To further investigate whether the WWTP core resistome was disseminated outside the treatment plant facilities, we mapped reads from online available metagenomes to the functionally selected WWTP resistome. These auxiliary metagenomes contained sequences from the human gut, cow rumen, permafrost and aquifer, in total more than two billions reads^37^,^38^,^39^,^40^. Notably, only six (8%) of the WWTP core resistance genes were found in these non-WWTP metagenomes, confirming that the WWTP core resistome is distinct from the resistomes of other environments (Fig. 4a). Rarefaction curve analysis of the non-WWTP metagenomes showed that the low identification frequency of the WWTP resistance genes was not due to undersampling (Supplementary Fig. 3). Four of the six genes found in the non-WWTP metagenomes had a nucleotide identity >95% to clinically relevant resistance genes, highlighting that a PCR-based approach would probably have successfully identified these genes. However, reliance solely on PCR or metagenomic read mapping to antibiotic resistance gene databases would have neglected the vast majority of the WWTP core resistome, which seems confined to the WWTP environment.

To explore whether the WWTP metagenomes contained important resistance genes, we compiled a list of 27 resistance genes commonly found in Gram-negative and Gram-positive pathogens^41^,^42^,^43^ (Supplementary Table 5). This list includes commonly encountered representatives of the *ctx-m* and *erm* gene families, as well as several important tetracycline resistance genes. A total of 578 sequence reads from the deeply sequenced AAW.5.2010 metagenome mapped to 10 of the resistance genes (Supplementary Fig. 4a). Nearly half of the reads (48%) mapped to the *tetA* resistance gene, whereas no reads mapped to the *ctx-m* genes. In contrast, a significantly higher number of 9,401 reads (*P*=0.008, Mann–Whitney *U*-test) mapped to a total of 53 of the 79 functionally selected resistance genes (Supplementary Fig. 4b). These mapping results confirm that clinically important resistance genes are indeed present in the WWTP environment, yet again they also highlight how reliance on targeted methodologies can be misleading when assessing the contribution of the WWTP core resistome to the dissemination of resistance genes outside of the WWTP environment.

As our study is focused on dissemination of the WWTP core resistome, we cannot rule out the possibility that low abundance species engage in important resistance gene exchange in WWTPs. Indeed, mobile plasmids carrying known resistance genes have been isolated from WWTPs^26^,^44^. However, our results show that only a small fraction of the WWTP core resistome participate in gene exchange, suggesting that WWTP microbiomes appear as probably as soil microbiomes to be sources for resistance gene dissemination. This conclusion should be cautioned by the fact that we only sampled WWTPs in one country. It is possible that these findings may be different in other parts of the world that deploy simpler WWTPs at higher temperatures and higher load of antibiotics.

An explanation for the limited dissemination of the WWTP core resistome could be that the resistance genes are not selected for or mobilized. However, studies have shown that even very low concentrations of antibiotics, probably present in WWTPs, can select for resistance determinants^45^,^46^. Therefore, we analysed the functionally selected inserts for the presence of canonical indicators of mobilization such as transposases and phage elements. We found that three (4%) of the functionally selected inserts carried canonical indicators of mobility including integrases associated with class I integrons, transposes and resolvases (Fig. 4b). Two of the resistance genes carried on these inserts (SPC_01 and AZI_01) had a high identity hit (>95% identity) to the NCBI nucleotide database. Furthermore, the resistance genes located on the AZI_01 and ERM_04 inserts could be found in the auxiliary metagenomes (Fig. 4a). This supports the hypothesis that mobilization is a major limiting factor for the dissemination of antibiotic resistance genes; for example, once a resistance gene is mobilized, it easily becomes disseminated across ecosystems. It should be noted that, owing to the relative short insert size (∼2 kb), some mobility elements may be overlooked. However, previous functional selections from human faecal samples using the same insert size found mobilization elements in 14% of the inserts, which is significantly higher than what is observed in this study (*P*=0.027, *χ*^2^-test)^12^.

Of particular interest was the spectinomycin resistance gene *aadA* in the SPC_01 insert. This gene was located in a class 1 integron and mapping of reads from the deeply sequenced metagenome to the insert containing the *aadA* gene showed that the integrase gene was roughly 50 times more abundant than the *aadA* gene, highlighting that the integron was not always associated with the *aadA* gene (Fig. 4c). This gene is part of the WWTP core resistome and has previously been found to be associated with wastewater environments including a French and a Chinese WWTP, effluent wastewater from a French hospital, pig manure sampled in Denmark and on a plasmid isolated from a Danish soil/manure sample^47^,^48^,^49^,^50^,^51^ (Supplementary Fig. 5). Interestingly, the gene had also been found in an *E. coli* isolate from a bloodstream infection (99.9% identity). This confirms that once mobilized, members of the core WWTP resistome can be found outside this environment and even in clinical isolates (Supplementary Fig. 5). Yet, only a small fraction of the core WWTP resistome has crossed this mobilization barrier.

## Discussion

Integrating metagenomic functional selection with deep metagenomic sequencing represents a powerful approach to tracing functionally validated antibiotic resistance genes across different environments. Using functional selections, we identified a core WWTP resistome consisting of mostly novel resistance genes that confer resistance to all antibiotics tested, highlighting that WWTPs is a reservoir of previously uncharacterized resistance genes.

16S rRNA sequencing of longitudinal samples collected over a 7-year period from the same WWTP revealed that the microbial community within the WWTP is remarkably stable. In addition, longitudinal metagenomic sequencing from the same WWTP demonstrated that the majority of the genes within the WWTP core resistome were also stably maintained within the WWTP. This indicates that the resistome is linked to the community, which was confirmed with procrustes analysis. Integration of metagenomic data sets from other WWTPs showed that a majority of the functionally verified resistance genes are also maintained in those microbial communities. These data point towards the existence of a shared WWTP core resistome harboured by the dominant members of the WWTP microbial community.

Notably, <10% of the WWTP core resistome sampled in this study is found in other metagenomic data sets or in sequences deposited at NCBI. Although database bias towards human pathogens and commensals could explain these findings, this also support that members of the WWTP core resistome rarely take part in gene exchange networks with human pathogens. Instead, the WWTP microbial community can be considered a unique and stable microbial community harbouring a rich resistome that is difficult to access by human pathogens or other bacteria. This conclusion is consistent with a previous metagenomic study based on read mapping to existing antibiotic resistance gene databases, showing that the WWTP resistance gene profiles are distinct from other environments^23^.

Our findings do not preclude the existence of clinically important resistance gene exchange in the WWTP. In fact, we do detect a subset of clinically important resistance genes in the deeply sequenced metagenome. Consequently, species found in low abundance in WWTPs, including human pathogens, may exchange resistance genes within this environment contributing to clinical problems of antibiotic resistance. However, our findings are consistent with the hypothesis that the abundant WWTP core resistome is only disseminated to a very limited extent to other microbial communities. The few resistance genes identified in this study to be disseminated to other microbial communities are associated with canonical mobility elements, suggesting that mobilization, rather than functionality, is the main barrier preventing spread of resistance genes.

Although our study demonstrates that the WWTP core resistome harbours an unappreciated diversity of resistance genes that are unique to this environment, the genetic diversity captured using functional selection is still limited, compared with the total genetic diversity within the WWTP environment. Consequently, the functional selection only allows us to analyse the more abundant resistance genes. Larger studies are needed to characterize the full WWTP resistome and its relation to clinical problems of antibiotic resistance. However, application of metagenomic methods such as those presented in this study should improve the risk classification of various environments, as it allows relating the number of clinically relevant resistance genes to the overall number of functional resistance genes. This will provide a useful tool for establishing control policies for environments that contribute substantially in the dissemination of antibiotic resistance genes.

## Methods

### DNA extraction

A total of 15 activated sludge samples were collected over a period of 3 years from five different Danish WWTPs with biological nitrogen and phosphorus removal. DNA was extracted using the FastDNA spin kit for soil (MP Biomedicals) according to the manufacturer's instructions, except an initial incubation for 3 min in 90 °C phenol. Briefly, 1 ml sample aliquots were centrifuged at 13,000 r.p.m. (17,000*g*) for 5 min and the supernatant discarded. The pellets were redissolved in 250 μl phosphate buffer (included in the FastDNA kit) and transferred to the FastDNA bead beating tubes. To each tube, 750 μl preheated phenol (Biological grade, pH 8, 0.1 M EDTA, Sigma-Aldrich) was added and the samples incubated for 3 min at 90 °C with occasional shaking. The samples were then bead beaten using the manufacturer's instructions and afterwards centrifuged at 13,000 r.p.m. for 10 min, to separate the phenol phase from the top liquid phase containing the DNA. The top phase was extracted and the DNA further purified using the FastDNA kit according to the manufacturer's instructions. DNA concentrations were measured using Qubit (Life Technologies) and DNA integrity evaluated using gel electrophoresis.

### Library for functional selection

DNA from WWTP AAW sampled in the third quarter of 2012 was used for functional selection. The DNA was sheared by Covaris E220 instrument (USA). Two hundred microlitres of DNA solution was sheared in a Covaris mini-tube according to Covaris shearing protocol for 2 kbp DNA fragments. Sheared DNA was end repaired using the End-It end repair kit (Epicentre) with the following protocol:

1. Combine:
  - 68 μl of sheared DNA (90 ng μl^−1^)
  - 10 μl 10× end repair buffer
  - 10 μl 2.5mM dNTP mix
  - 10 μl 10mM ATP
  - 2 μl of End It enzyme mix.
2. Incubate at room temperature for 45 min.
3. Stop reaction by heating at 70 °C for 20 min.

End-repaired DNA was size selected by electrophoresis through a 1% low melting point agarose gel in 0.5 × Tris-Borate-EDTA (TBE) buffer. A gel slice corresponding to 1,500–3,000 bp was excised from the gel and DNA was extracted using a QIAquick Gel Extraction Kit (Qiagen). DNA was ligated into pZE21 at the HincII site using the Fast Link ligation kit (Epicenter) with the following ligation protocol:

1. Combine:
  - 1.0 μl 10× fast ligation buffer
  - 0.5 μl 10mM ATP
  - 1.0 μl HincII-cut pZE21 MCS 1 vector (65 ng μl^−1^)
  - 2.50 μl sheared, end-repaired, gel-purified DNA insert (154 ng μl^−1^)
  - 1.0 μl Fast-Link DNA ligase (2 units per μl).
2. Incubate at room temperature for 16 h.
3. Heat inactivate at 70 °C for 20 min.

Then, 3 μl of the fresh ligation mixture was used for electro-transformation (Btex electroporator) into 50 μl of electrocompetent *E. coli* TOP10 cells (Invitrogen). After transformation using standard protocols for a 2-mm electroporation cuvette (Biorad), cells were recovered in 1 ml super optimal broth with catabolite repression (SOC) medium for 1 h at 37 °C. Libraries were titred by plating out 1, 0.1 and 0.01 μl of recovered cells onto lysogeny broth (LB) agar plates containing 50 μg ml^−1^ kanamycin. For each library, insert size distribution was estimated by gel electrophoresis of colony PCR products obtained by amplifying the insert using primers flanking the HincII site of the multiple cloning site of the pZE21 MCS1 vector (which contains a selectable marker for kanamycin resistance). The average insert size for all libraries was found to be 2.07 kbp. The total size of metagenomic library was 8.28 × 10^8^ bp as determined by multiplying average PCR-based insert size with the number of colony forming units. The rest of the recovered cells were inoculated into 10 ml of LB containing 50 μg ml^−1^ kanamycin and grown overnight. The overnight culture was frozen down with 15% glycerol and kept at −70 °C for subsequent screening.

### Functional selections of antibiotic-resistant clones from metagenomic library

100 μl of the insert library freezer stock corresponding to 5 × 10^6^ colony forming units, were plated out on LB agar plates containing binary combinations of kanamycin (50 μg ml^−1^) and 1 of 15 different antibiotics (Table 1), and incubated at 37 °C for 16 h. Assay drug concentrations were chosen to prevent growth of overnight control libraries with the empty vector. Based on the original library titres as well as titres of the freezer stocks, the average amplification of a given library can be estimated. On average, each unique clone in the library was plated out with 10 × coverage. To minimize the redundancy of clones in subsequent analysis, on average, the number of colonies picked for sequencing corresponded to approximately the number of resistant clones on an agar plate divided by the estimated average library amplification.

Each of the clones picked was inoculated into 96 deep-well plates containing liquid LB medium supplemented with kanamycin (50 μg ml^−1^) and the antibiotic to which resistance had been selected (Table 1), and grown overnight to verify resistance phenotype.

### Sequencing and analysis of metagenomic inserts

Functional selected metagenomic inserts were sequenced using Sanger sequencing. A total of 749 clones were sequenced bidirectionally using the following primers:

Forward primer_pZE21_81_104_57C: 5′- GAA TTC ATT AAA GAG GAG AAA GGT -3′

Reverse primer_pZE21_151_174rc_58C: 5′- TTT CGT TTT ATT TGA TGC CTC TAG -3′.

All reads (⩾500 bp) obtained after sequencing were assembled into contigs using CLC Main Workbench 6. The sequence from each contig corresponds to single clone containing unique insert. The full insert sequence for each unique insert was obtained by merging the forward primer sequence reads and reverse primer sequence reads using EMBOSS:Merger server (bioinfo.nhri.org.tw/cgi-bin/emboss/merger, accessed: 20 March 2013). Open reading frames were identified and annotated using ORFfinder (http://www.ncbi.nlm.nih.gov/projects/gorf/, accessed: 20 April 2013). Annotated resistance genes were compared with the GenBank non-redundant nucleotide database (accessed: 1 May 2013) using tblastx, which computes local sequence alignment between the nucleotide query translated in all six frames and the non-redundant nucleotide database translated in all six frames. For each query, the genbank ID and the alignment coordinate for the top scoring tblastx hit was obtained. For each of these top-scoring hits, any annotated feature that overlapped the alignment coordinates in the specific Genbank file was obtained from GenBank. Global sequence alignments and corresponding percentage identities between the query and the obtained sequences were computed using EMBOSS:Stretcher at the nucleotide level in the annotated frame. When multiple annotated features were obtained for a query sequence, only the sequence most similar to the query at the nucleotide level was retained (Supplementary Table 1).

### Metagenome sequencing

Samples were prepared for sequencing using the Nextera DNA Sample Preparation Kits (Illumina Inc.) with 50 ng of DNA. The library DNA concentration was measured using the QuantIT kit (Molecular Probes) and paired-end sequenced (2 × 151 bp) on an Illumina HiSeq2000 using the TruSeq PE Cluster Kit v3-cBot-HS and TruSeq SBS kit v.3-HS sequencing kit (Illumina Inc.). The sample AAW-2012-3 was paired-end sequenced (2 × 301 bp) on the Illumina MiSeq platform using the MiSeq Reagents kit v2 (Illumina Inc.) (Supplementary Table 4).

### Metagenome read trimming and mapping

Metagenome reads in fastq format were imported to CLC Genomics Workbench v. 7.03 (CLC Bio) and trimmed using a minimum phred score of 20, a minimum length of 50 bp, allowing no ambiguous nucleotides and trimming off Illumina Nextera sequencing adapters, if found. The trimmed metagenome reads were mapped to all antibiotic contigs using CLC Genomics Workbench v. 7.03, using a minimum of 95% similarity over the full read length. The number of reads hitting within the putative antibiotic gene were counted and further data analysis and visualization was conducted using R^52^.

The trimmed metagenome reads were also mapped to the Greengenes 16S rRNA database version 13_5 using the map reads to reference function in CLC genomics Workbench v. 7.03 requiring 70% similarity over the full read length and random assignments of reads, which mapped to two sequences equally well.

### Metagenome *de novo* assembly

The trimmed metagenome reads from the AAW-5-2010 sample were assembled using CLC's *de novo* assembly algorithm, using a kmer of 63 and a minimum scaffold length of 2 kbp.

### Metagenomes from other environments

Metagenome reads from four other environments, human gut^37^ (ERS006497), permafrost^38^, cow rumen^39^ (SRP004875) and an aquifer^40^ (SRA050978), were downloaded from the NCBI SRA and used for comparison. Trimming and read mapping to the antibiotic contigs were performed as previously described.

### 16S rRNA gene sequencing

Activated sludge was sampled from aeration tanks at AAW WWTP. Sampling were carried out up to four times a year from 2006 to 2013, resulting in a total of 24 samples.

DNA extraction was conducted using the FastDNA spin kit for soil (MP Biomedical) according to the manufacturer's instructions, except the bead beating was increased to 4 × 40 s at 6 m s^−1^ using a FastPrep FP120 (MP Biomedicals). The procedure for bacterial 16S rRNA amplicon sequencing targeting the V1-3 variable region was modified from ref. 53. Briefly, 10 ng of extracted DNA was used as template and the PCR reaction (25 μl) contained dNTPs (400 nM of each), MgSO_4_ (1.5 mM), Platinum Taq DNA polymerase HF (2 mU), 1 × Platinum High Fidelity buffer (Thermo Fisher Scientific) and a pair of barcoded library adaptors (400 nM), V1-3 primers 27F: 5′- AGAGTTTGATCCTGGCTCAG -3′ and 534R: 5′- ATTACCGCGGCTGCTGG -3′. Thermo cycler settings: initial denaturation at 95 °C for 2 min, 30 cycles of 95 °C for 20 s, 56 °C for 30 s, 72 °C for 60 s and final elongation at 72 °C for 5 min. All PCR reactions were run in duplicate and pooled afterwards. The amplicon libraries were purified using the Agencourt AMpure XP bead protocol (Beckmann Coulter, Brea, CA, USA) with the following exceptions: the sample/bead solution ratio was 5/4 and the purified DNA was eluted in 33 μl nuclease-free water. Library concentration was measured with Quant-iT HS DNA Assay (Thermo Fisher Scientific) and quality validated with a Tapestation 2200 using D1K ScreenTapes (Agilent). Based on library concentrations and calculated amplicon sizes, the samples were pooled in equimolar concentrations and diluted to 4 nM. The library pool was sequenced on a MiSeq (Illumina, San Diego, CA, USA) using a MiSeq Reagent kit v3 (2 × 300 PE) following the procedure in ref. 54, with exception of 10% phiX control library (Illumina) spike-in and final library loading concentration of 20 pM. A detailed DNA extraction and amplicon sequencing protocol can be obtained from www.midasfieldguide.org/en/protocols.

### 16S rRNA data processing

All sequenced sample libraries were subsampled to 50,000 raw reads and low-quality reads removed using Trimmomatic v. 0.32 (ref. 55) with the settings SLIDINGWINDOW:1:3 and MINLEN:275. All high-quality PE reads were screened for phiX contamination using bowtie2 v. 2.1.0 (ref. 56), with standard settings and all matching reads removed. The potential phiX contamination is due to the use of an un-indexed phiX as a quality control, which can result in index carryover from nearby clusters with indexes.

Forward and reverse reads were merged using FLASH v. 1.2.7 (ref. 57), with the setting -r 300 -f 475 -s 50 -m 25 -M 200 and afterwards merged reads smaller than 425 bp or larger than 525 bp were discarded.

The merged reads were de-replicated and formatted for use in the uparse workflow^58^. The merged reads were clustered using the usearch v. 7.0.1090–cluster_otus with default settings. Operation taxonomic units (OTU) abundance was estimated using the usearch v. 7.0.1090–usearch_global with–id 0.97. Taxonomy was assigned using the RDP classifier^59^ as implemented in the parallel_assign_taxonomy_rdp.py script in QIIME^53^ using the Greengenes database version 13_5 (ref. 60).

### 16S rRNA data analysis and visualization

All data analysis and visualizations were conducted using R^52^ through the Rstudio IDE (http://www.rstudio.com/). Briefly, OTU counts and associated taxonomic assignments were imported and merged to a phyloseq object^61^. All samples were rarefied to 10,000 reads and subsequently OTUs with a maximum count of <5 in the 24 samples were removed.

Community stability within WWTP AAW as a function of time was calculated by comparing Bray–Curtis β-diversity between all samples.

### Rarefaction curve analysis

The rarefaction curves are based on the number of reads that map (>95% identity and >95% coverage) to the functional selected resistance genes. The curves are computed using the R package Vegan (step size=1 × 10^6^) and ggplot2 (refs 62, 63). Abundance estimates are calculated using the Vegan implementation of the species accumulation model described by Chiu *et al*.^64^.

### BLAST of functionally selected resistance genes

Functionally selected resistance genes from WWTP AAW, human gut^12^,^32^ and soil^33^,^34^,^35^,^36^ were blasted against NCBI genbank nt database, excluding self-hits. The top hit was aligned to the functionally selected resistance gene using EMBOSS Matcher and the identity was calculated^65^.

### Mapping to clinically relevant genes

The trimmed metagenome reads were mapped to reference database (Supplementary Table 1) using CLC Genomics Workbench v. 7.03, using a minimum of 95% similarity over the full read length. The number of reads hitting within the putative antibiotic gene were counted, and further data analysis and visualization were conducted using R.

### Procrustes analysis

Square-root-transformed metagenome abundance counts of the antibiotic resistance genes were used for a principal component analysis (PCA) analysis in R through the vegan package^62^. For comparison, matching samples (for a few samples, the closest time point had to be used as matching samples were not available) from the larger 16S rRNA data set were extracted and square-root-transformed OTU abundance counts used for a PCA analysis. The two PCAs were compared using the procrustes function implemented in vegan and significance tested using the protest function with 999 permutations.

## Additional information

**Accession codes:** Nucleotide sequences of the functionally selected resistance genes have been deposited in the Genbank database with accession codes KT387137 to KT387215. The raw metagenome reads have been deposited in the European Nucleotide Archive under study PRJEB8087. The assembled metagenome scaffolds from AAW.5.2010 have been deposited in the MG-RAST database with accession code MG-RAST4611649.3. The raw V13 16S rRNA amplicon sequences are part of the MiDAS data set (www.midasfieldguide.org) and have been deposited in the European Nucleotide Archive under study PRJEB8105. The accession codes for the specific samples used are: ERS634988, ERS634989, ERS634990, ERS634991, ERS635004, ERS635036, ERS635037, ERS635038, ERS635362, ERS635433, ERS635452, ERS635453, ERS635454, ERS635455, ERS635477, ERS635478, ERS635521, ERS635529, ERS635530, ERS635539, ERS635540, ERS635541, ERS635549 and ERS635550.

**How to cite this article:** Munck, C. *et al*. Limited dissemination of the wastewater treatment plant core resistome. *Nat. Commun.* 6:8452 doi: 10.1038/ncomms9452 (2015).

## Supplementary Material

Supplementary Information Supplementary Figures 1-5 and Supplementary Tables 1-5

## Figures and Tables

**Table 1:** Selection conditions.

**Antibiotic**	**Concentration (μg** **ml**^−1^)	**Code**	**Class**	**Number of colonies**
Ampicillin	16	AMP	β-Lactam/penicillin	660
Amoxicillin	16	AMX	β-Lactam/penicillin	500
Carbenicillin	64	CAR	β-Lactam/penicillin	1,100
Piperacillin	16	PIP	β-Lactam/penicillin	800
Ceftazidime	1	CTZ	β-Lactam/cephalosporin	350
Amikacin	16	AMK	Aminoglycoside	220
Gentamicin	8	GEN	Aminoglycoside	40
Spectinomycin	32	SPC	Aminoglycoside	330
Azithromycin	16	AZI	Macrolide	120
Erythromycin	100	ERY	Macrolide	200
Tetracycline	4	TET	Tetracycline	100
Chloramphenicol	6	CAM	Phenicol	30
Rifampicin	16	RIF	Rifamycin	210
Trimethoprim	4	TMP	Benzylpyrimidine	2,000
Sulfamethoxazole/trimethoprim	32:4	SXT	Sulfonamide/dihydrofolate reductase inhibitor	1,500
Total				8,160

AMK, amikacin; AMP, ampicillin; AMX, amoxicillin; AZI, azithromycin; CAM, chloramphenicol; CAR, carbenicillin; CTZ, ceftazidime; ERY, erythromycin; GEN, gentamicin; PIP, piperacillin; RIF, rifampicin; SPC, spectinomycin; SXT, sulfamethoxazole/trimethoprim; TET, tetracycline; TMP, trimethoprim.
