# Supplementary material for: Limited dissemination of the wastewater treatment plant core resistome
Source: Nat Commun. 2015 Sep 30;6:8452. doi: 10.1038/ncomms9452 (PMC4598724; doi:10.1038/ncomms9452)
Supplement: Supplementary Information — Supplementary Figures 1-5 and Supplementary Tables 1-5 [file ncomms9452-s1.pdf]

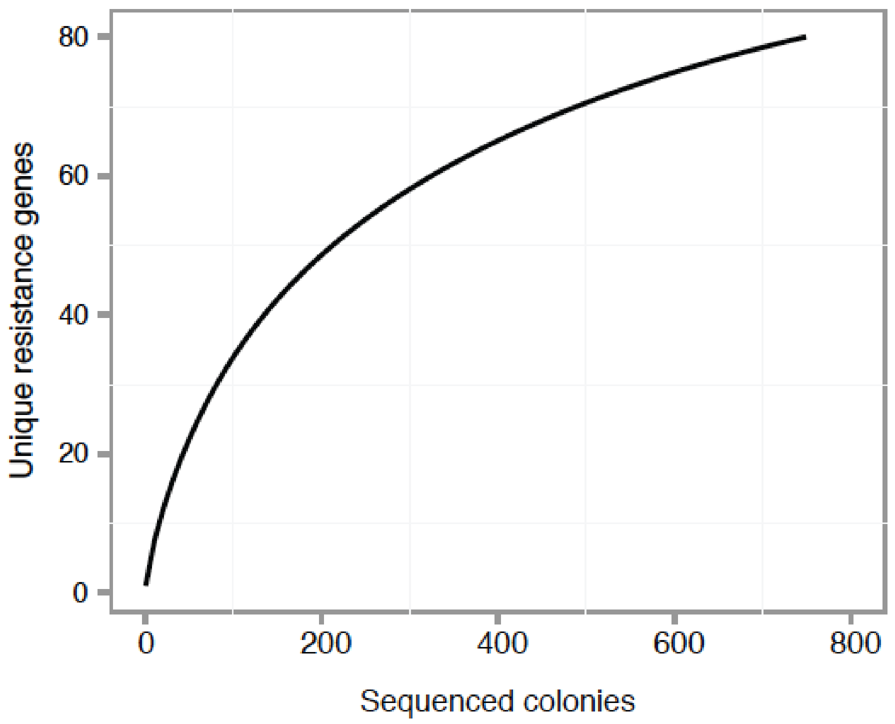

Supplementary Figure 1 | Sampling completeness
Rarefaction curve showing the increase of unique colonies as function of the number of sequenced colonies from the functional selection. In total 749 colonies were sequenced from the different selection plates (Table 1 and Methods) resulting in 79 unique resistance genes. The curve indicates that the majority of the resistance genes in the WWTP sample were identified.

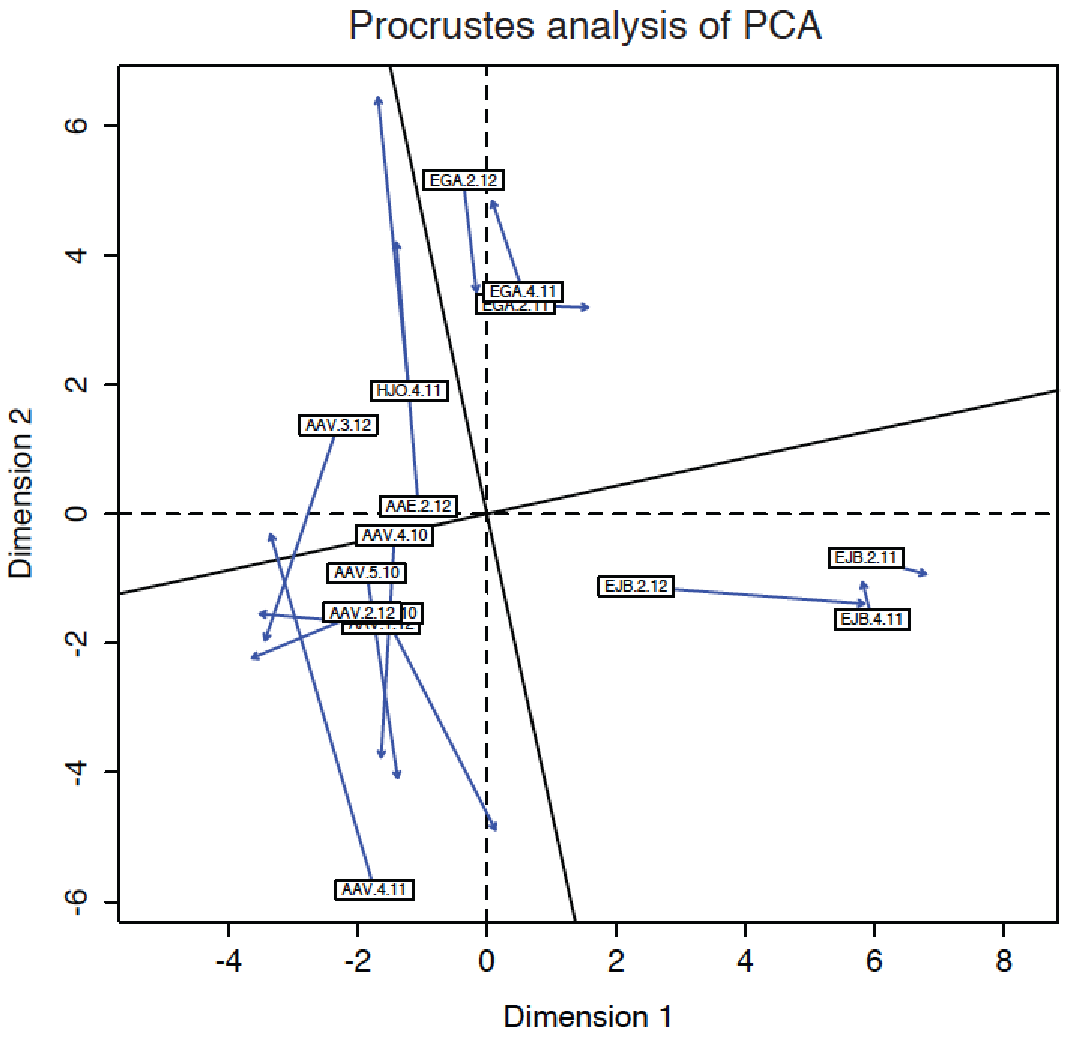

Supplementary Figure 2 | Procrustes analysis
Procrustes analysis of the correlation between the principle component analysis (PCA) of the metagenome abundance counts of the antibiotic resistance genes and the PCA of the 16S rRNA abundances (Methods). The analysis reveals a significant ($p < 0.001$) correlation between the 16S community profile and the resistance gene profile in the different WWTP samples, supporting the hypothesis that the resistome is shaped bacterial phylogeny.

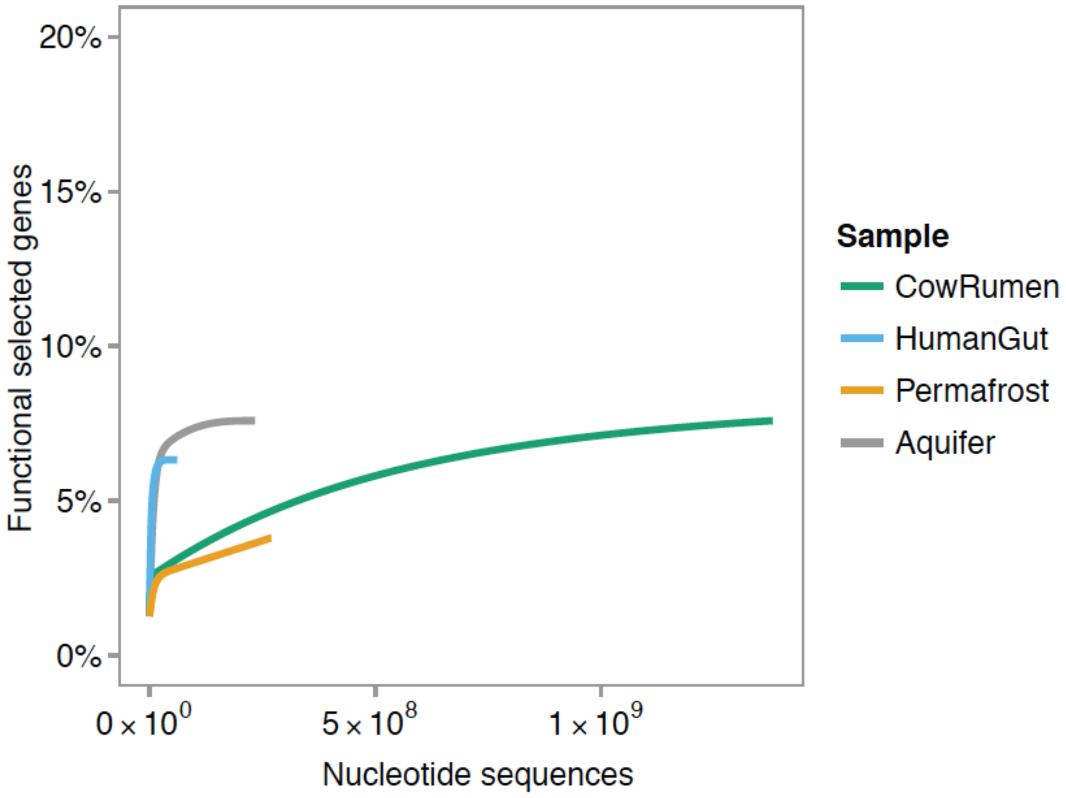

Supplementary Figure 3 | Rarefaction curve of non-WWTP
Mapping data from the auxiliary metagenomes was used to generate rarefaction curves that show the number of unique WWTP resistance genes found in the non-WWTP metagenomes as a function of the sample size. The read mapping parameters used was >95 % identity and >95 % coverage (Methods).

a

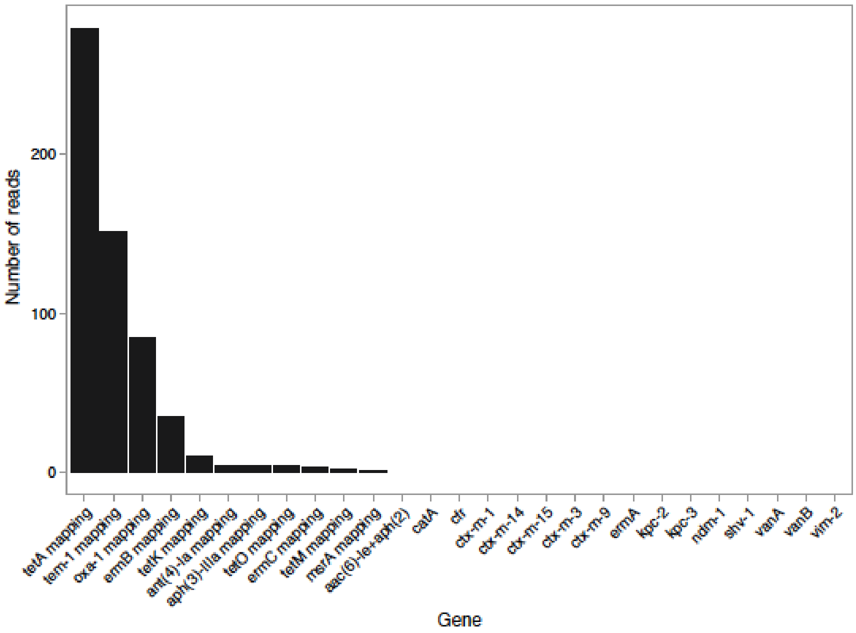

b

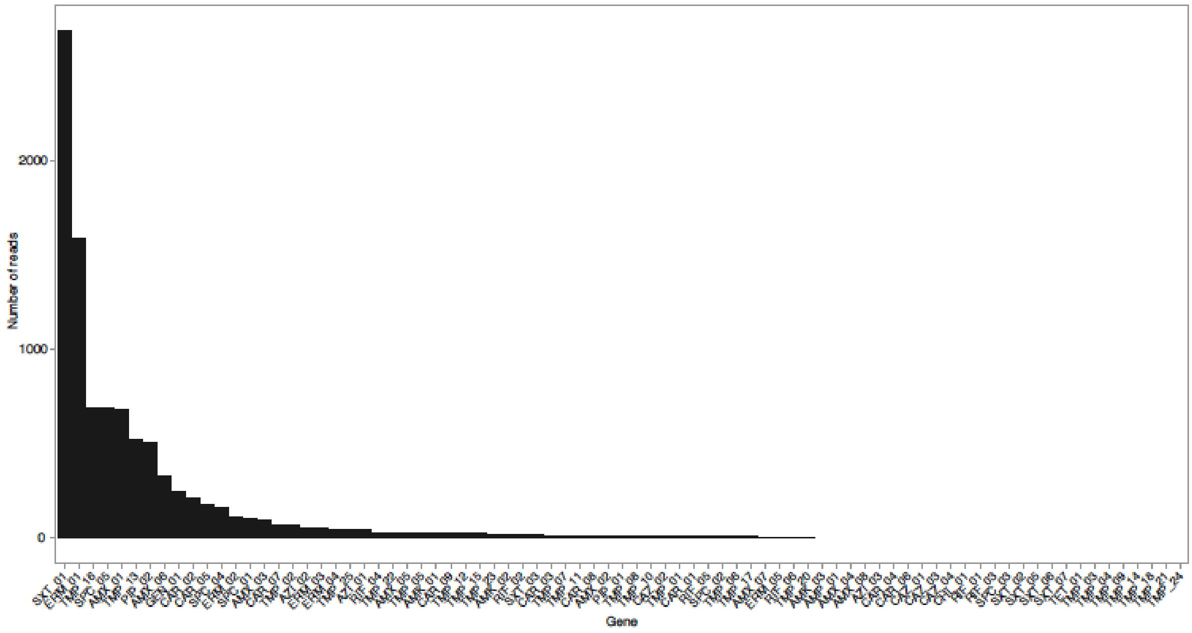

Supplementary Figure 4 | Mapping of metagenomic form AAW.5.2010

The 1.1 billion sequence reads from the deeply sequenced WWTP metagenome AAW.5.2010 were mapped to set of 29 clinically relevant resistance genes (a) and to the functionally selected resistance genes (b). Successful mapping required a minimum of 95 % identity and 95 % coverage.

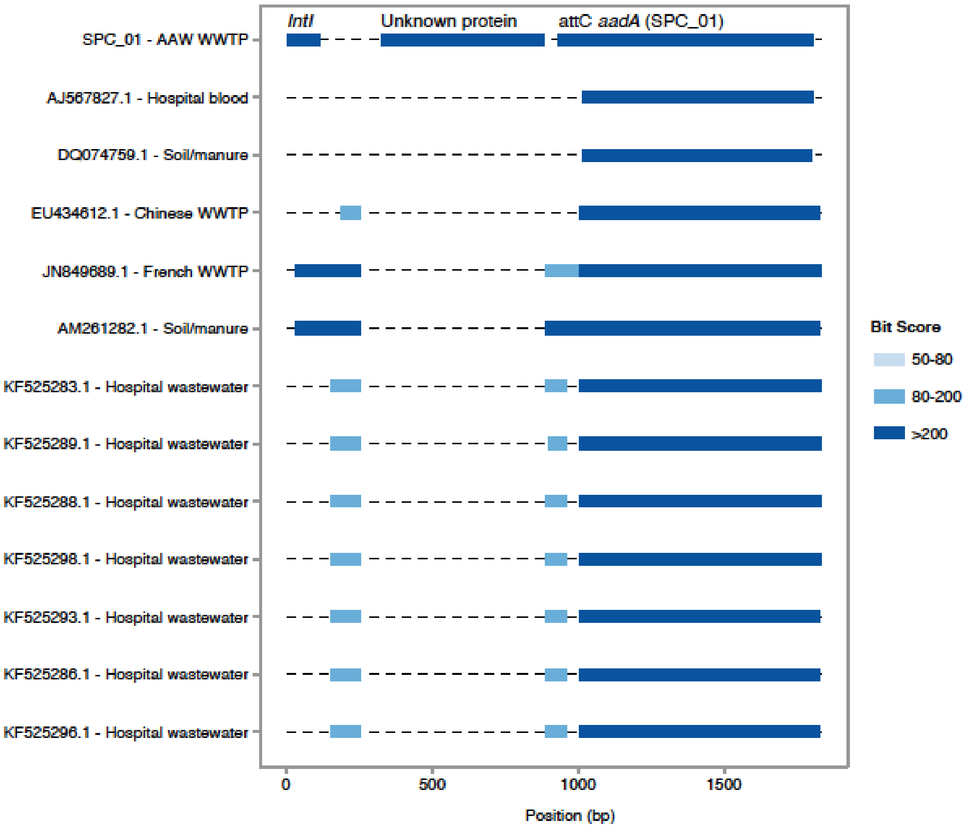

Supplementary Figure 5 | SPC_01 BLAST
The functionally selected insert containing the SPC_01 gene was blasted against the genbank nt database. The result shows hits where the identity to the SPC_01 resistance gene (aadA) was >= 95 %. Each result is denoted by its accession number and isolation source. Interestingly this gene seems to be associated with the wastewater environment, with one example of the gene appearing in a clinical sample.

Supplementary Table 1| WWTP information

	Fraction of industrial wastewater*	Load (PE)	Receiving hospital wastewater
Aalborg East	10	45000	No
Aalborg West	30	195000	Yes
Egaa	30	84000	Yes
Hjoerring	30	100000	Yes
Ejby	55	286000	Yes

PE: Population equivalent

*Calculated as approximate percentage of total organic load measured as chemical oxygen demand (COD)

Suppplementary Table 2| The functional selected resistance genes

Antibiotics	Gene ID	Gene length (bp)	Gene annotations	Top hit	Genbank % ID
Carbenicillin	CAR_01	507	Beta-lactamase	JN559393.1|Pseudomonas aeruginosa |8651-9454	56.1
	CAR_02	1005	Beta-lactamase	FN640464.1|Uncultured bacterium lpxB gene for putative lipid-A-disaccharide synthase and bla gene for beta-lactamase class A, clone Ap6-8w|1523-2482	55.9
	CAR_03	909	Beta-lactamase	CP002859.1|Runella slithyformis DSM 19594|5147053-5147949	66.2
	CAR_04	798	NDM metallo-beta-lactamase	JN104597.1|E. coli strain EC405|115-927	57.2
	CAR_05	861	Beta-lactamase	JQ624676.1|Mammalian expression vector pSA95|4195-5055	100
	CAR_06	900	Beta-lactamase	GU441460.1|E. coli strain R170 plasmid pRZA92|3220-4125	60
	CAR_07	480	Beta-lactamase	CP000383.1|Cytophaga hutchinsonii ATCC 33406| 2205559-2206377	38.1
	CAR_08	825	OXA-2 beta-lactamase	JX846494.1|Pseudomonas aeruginosa strain Pa314 Class I integron OXA-2 like protein|109-909	97.1
	CAR_09	882	Beta-lactamase	CP000450.1|Beta-lactamase Nitrosomonas eutropha C91| 1096410-1097369	80.7
Piperacillin	PIP_01	771	Beta-lactamase	CP002961.1|Emticicia oligotrophica DSM 17448|1889449-1890237	
	PIP_02	816	Beta-lactamase class D	CP000383.1|Cytophaga hutchinsonii ATCC 33406| 2205559-2206377	62.6
Ceftazidime	CTZ_01	759	Beta-lactamase	CP002961.1|Emticicia oligotrophica DSM 17448|870369-871124	70.5
	CTZ_02	768	Beta-lactamase	CP002961.1|Emticicia oligotrophica DSM 17448| 870369-871124	69.7
	CTZ_03	732	Metallo-beta-lactamase	FP476056.1|Zobellia galactanivorans| 970040-970783	58.1
	CTZ_04	768	Hypothetical protein	CP003787.1|Riemerella anatipestifer RA-CH-1| 30846-31760	100
Amoxicillin	AMX_01	651	Beta-lactamase	CP000356.1|Sphingopyxis alaskensis RB2256|2002725-2003594	80
	AMX_02	915	Beta-lactamase	CP002859.1|Runella slithyformis DSM 19594|5147053-5147949	67.8
	AMX_03	975	Beta-lactamase	CP001220.1|Comamonas testosteroni CNB-2|2966741-2967490	57.9
	AMX_04	762	Beta-lactamase	CP000361.1|Arcobacter butzleri RM4018|1485957-1486718	81.2
	AMX_05	417	Beta-lactamase	CP002859.1|Runella slithyformis DSM 19594|5147053-5147949	32.4
	AMX_06	915	Beta-lactamase like protein	CP000248.1|Novosphingobium aromaticivorans DSM 12444|1837704-1838573	56.2
	AMX_07	858	Penicillin binding protein transpeptidase	CP002961.1|Emticicia oligotrophica DSM 17448|2038928-2039734	62.9
	AMX_08	846	Beta-lactamase	CP000269.1|Janthinobacterium sp. Marseille|647835-648734	46.4

Ampicillin	AMP_01	1020	Beta-lactamase	AP012047.1|Arcobacter butzleri ED-1 DNA|1416170-1416931	60.3
Spectinomycin	SPC_01	792	Aminoglycoside adenylyltransferase	JN849689.1|Uncultured bacterium plasmid pRSB113|6315-7106	100
	SPC_02	1005	Spectinomyin phosphotransferase	FN650140.1|Legionella longbeachae NSW150|1157668-1158690	56.3
	SPC_03	801	Spectinomycin phosphotransferase	FN650140.1|Legionella longbeachae NSW150|1157668-1158690	26.9
	SPC_04	1014	Aminoglycoside -(3")(9)-adenylyltransferase	FJ172373.1|Uncultured bacterium clone BF7_C6 class 1 integron qacH and aadA genes|598-1443	56.7
	SPC_05	1092	Aminoglycoside phosphotransferase	CP001824.1|Sphaerobacter thermophilus DSM 20745| 940698-941714	50.7
Gentamicin	GEN_01	636	GCN5-like N-acetyltransferase	CP002447.1|Mesorhizobium ciceri biovar biserrulae WSM1271| 525402-525917	59.3
Amikacin	AMK_01	339	Aminoglycoside 6'-N-acetyltransferase	AY566824.1|Uncultured soil bacterium clone CR6 putative glucosamine-fructose-6-phosphate aminotransferase gene, partial cds; aminoglycoside 6'-N-acetyltransferase gene|249-797	45.2
	AMK_02	567	Aminoglycoside 6'-N-acetyltransferase	AY566824.1|Uncultured soil bacterium clone CR6 putative glucosamine-fructose-6-phosphate aminotransferase gene, partial cds; aminoglycoside 6'-N-acetyltransferase gene|249-797	66.4
	AMK_03	501	Aminoglycoside 6'-N-acetyltransferase	AY566820.1|Uncultured soil bacterium clone 85C1 putative cation efflux family protein and aminoglycoside 6'-N-acetyltransferase genes|354-905	65.9
Chloramphenicol	CAM_01	1428	Chloramphenicol resistant protein	KC176455.1|RNAi silencing vector pCAPD|3580-4239	46.2
Tetracycline	TET_01	1191	Putative tetracycline resistant protein	CP003504.1|Enterococcus hirae ATCC 9790|2237403-2238605	54.1
Azithromycin	AZI_01	759	Ribosomal RNA adenine dimethylase family protein	FR720602.1|Streptococcus oralis Uo5| 1839657-1840394	96.8
	AZI_02	777	rRNA (adenine N-6-)-methyltransferase	CP001778.1|Stackebrandtia nassauensis DSM 44728| 2735336-2736172	53.9

	AZI_03	618	MscS mechanosensitive ion channel	CP002084.1|Dehalogenimonas lykanthroporepellens BL-DC-9|1509935-1510822	40.5
Erythromycin	ERM_01	399	Dimethyl adenine transferase	CP001686.1|Kytococcus sedentarius DSM 20547| 1860415-1861149	35.3
	ERM_02	636	GTP binding protein Hflx	CP002876.1|Nitrosomonas sp. Is79A3|1604493-1605845	43.6
	ERM_03	438	rRNA(adenine N-6-)- methyltransferase	CP001778.1|Stackebrandtia nassauensis DSM 44728| 2735336-2736172	31.8
	ERM_04	405	rRNA (adenine N-6-)-methyltransferase	CP003922.1|Streptococcus suis SC070731|	53.1
	ERM_05	513	Macrolide-efflux protein	EU870852.1|Streptococcus pyogenes strain MB56Spyo005|1-1227	40.8
Trimethoprim	TMP_01	306	Dihydrofolate reductase	CP000267.1 |Rhodoferax ferrireducens T118| 3291945-3292439	40.7
	TMP_02	696	Thymidylate synthase	CP002419.1 |Neisseria meningitidis G2136| 1675708-1676502	59.2
	TMP_03	408	Thymidylate synthase	CP001681.1 |Pedobacter heparinus DSM 2366| 1689195-1689680	
	TMP_04	489	Dihydrofolate reductase	CP002046.1 |Croceibacter atlanticus HTCC2559| 2891530-2892012	57.1
	TMP_05	495	Dihydrofolate reductase	CP000449.1 |Maricaulis maris MCS10| 2207968-2208489	61
	TMP_06	501	Dihydrofolate reductase	CP002691.1 |Haliscomenobacter hydrossis DSM 1100| 4468410-4468913	58.9
	TMP_07	489	Dihydrofolate reductase	CP003178.1 |Niastella koreensis GR20-10| 5722263-5722763	61.8
	TMP_08	495	Putative oxidoreductase	AP012337.1 |Caldilinea aerophila DSM 14535| 4963388-4964050	41.4
	TMP_09	468	Dihydrofolate reductase	CP000089.1 |Dechloromonas aromatica RCB| 646778-647269	58.9
	TMP_10	537	Dihydrofolate reductase	HE965806.1 |Bordetella bronchiseptica 253| 2778656-2779153	51.4
	TMP_11	432	Thymidylate synthase	CP003418.1 |Ignavibacterium album JCM 16511| 2413030-2413917	76.4
	TMP_12	510	Dihydrofolate reductase	CP002281.1 |Ilyobacter polytropus DSM 2926| 545109-545585	48.3
	TMP_13	762	dihydrofolate reductase	CP001013.1 |Leptothrix cholodnii SP-6| 1345906-1346406	43.3
	TMP_14	480	Dihydrofolate reductase	CR954246.1 |Pseudoalteromonas haloplanktis str. TAC125 |2808343-2808810	54.4
	TMP_15	816	Thymidylate synthase	CP002542.1 |Fluviicola taffensis DSM 16823| 960816-961610	70.4
	TMP_16	525	Dihydrofolate reductase	CP001339.1 |Thioalkalivibrio sulfidophilus HL-EbGr7|2890732-2891220	58.2
	TMP_17	483	Dihydrofolate reductase	CP001638.1|Geobacillus sp. WCH70|1654473-1654961	55
	TMP_18	492	Dihydrofolate reductase	CP000148.1 |Geobacter metallireducens GS-15| 3337712-3338197	60.8
	TMP_19	483	Dihydrofolate reductase	CU207366.1 |Gramella forsetii KT0803| 328588-329070	69.2
	TMP_20	753	Putative oxidoreductase	AP012337.1 |Caldilinea aerophila DSM 14535| 4963388-4964050	48.3

Sulfamethoxazole / Trimethoprim	TMP_21	663	Bifunctional deaminase/reductase protein	CP002040.1 |Nocardiopsis dassonvillei subsp. dassonvillei DSM 43111| 3369988-3370797	39.6
	TMP_22	516	Dihydrofloate reductase	CP000316.1 |Polaromonas sp. JS666| 1886763-1887257	62.6
	TMP_23	525	Dihydrofloate reductase	CP000747.1 |Phenylobacterium zucineum HLK1| 902351-902869	60.7
	TMP_25	495	Thymidylate synthase	FQ859181.1 |Hyphomicrobium sp. MC1|3728475-3729269	38.2
	SXT_01	795	Thymidylate synthase	FO082820.1 |Rhizobium sp. str. NT-26|2040923-2041717	71.9
	SXT_02	687	Thymidylate synthase	CP000284.1 |Methylobacillus flagellatus KT| 953119-953913	52.2
	SXT_03	444	Thymidylate synthase	JF924881.1 |Uncultured bacterium clone tri1|390-794	65.1
	SXT_05	414	Thymidylate synthase	HE774682.1 |Flavobacterium indicum GPTSA100-9| 562831-563655	33.5
	SXT_06	831	Thymidylate synthase	CP000082.1 |Psychrobacter arcticus 273-4| 2477380-2478282	48.7
	SXT_07	483	Dihydrofolate reductase	CU207366.1 |Gramella forsetii KT0803| 328588-329070	69.2
Rifampicin	RIF_01	429	Rifampin ADP-ribosyl transferase	JX875536.1|Uncultured bacterium clone WGRif3028|3134-3583	72.3
	RIF_02	582	Pentapeptide repeat protein	CP002542.1|Fluviicola taffensis DSM 16823| 847426-847995	59.1
	RIF_03	426	Rifampin ADP-ribosyl transferase	HE577629.1|Vibrio splendidus partial integrative and conjugative element ICEVspPor2| 7798-8245	62.9
	RIF_04	744	Rifampin ADP-ribosyl transferase	BA000045.2|Gloeobacter violaceus PCC 7421|4141456-4141998	55.7
	RIF_05	750	Rifampin ADP-ribosyl transferase	FJ418586.4|Oscillatoria sp. PCC 6506 cylindrospermopsin|	72
	RIF_06	447	Rifampin ADP-ribosyl transferase	CP002859.1|Runella slithyformis DSM 19594| 5150381-5150830	74.9

Supplementary Table 3| Sampling and sequencing depth for the 15 metagenomes

Treatment plant	Year	Quarter	Sequencing platform	Trimmed reads (millions)
AAW	2012	3	MiSeq, 2x301	29
AAW	2012	2	HiSeq2000, 2x151	66
AAW	2012	1	HiSeq2000, 2x151	59
AAW	2011	4	HiSeq2000, 2x151	130
AAW	2010	5	HiSeq2000, 2x151	1109
AAW	2010	4	HiSeq2000, 2x151	24
AAW	2010	2	HiSeq2000, 2x151	113
AAE	2012	2	HiSeq2000, 2x151	151
HJO	2011	4	HiSeq2000, 2x151	94
EJB	2012	2	HiSeq2000, 2x151	91
EJB	2011	4	HiSeq2000, 2x151	127
EJB	2011	2	HiSeq2000, 2x151	90
EGA	2012	2	HiSeq2000, 2x151	59
EGA	2011	4	HiSeq2000, 2x151	137
EGA	2011	2	HiSeq2000, 2x151	78

Aalborg West (AAW), Aalborg East (AAE), Hjoerring (HJO), Ejby (EJB) and Egaa (EGA). Quarter "5" refers to December.

Supplementary Table 4| Contigs BLAST against NCBI nt database

Resistance Gene	ContigID	Contig length	Organism	Query cover	E value	Identity	Accession
CAR_05	81434	2.3 kb	Expression vector	97%	0	99%	AB235904.1
AMX_06	8037	68.8 kb	Sphingopyxis alaskensis	61%	0	76%	CP000356.1
CAR_07, PIP_02	25586	15.9 kb	Oceanithermus profundus	25%	0	73%	CP002361.1
ERM_01	10632	6.8 kb	Streptomyces albus	24%	0	71%	CP004370.1
TMP_25	363550	6.9 kb	Uncultured bacterium clone S07_TR_70	13%	2.00E-156	77%	KJ693103.1
TMP_13, TMP16	13117	4.9 kb	Propionibacterium acidipropionici	12%	6.00E-35	83%	CP003493.1
CAR_02	91841	53.5 kb	Pseudomonas stutzeri	4%	1.00E-126	70%	CP007441.1
AZT_02, ERM_03	204726	4.3 kb	Ilumatobacter coccineus	2%	8.00E-21	81%	AP012057.1

Contigs assembled from sample AAW.5.2010 that carried functional selected resistance genes were blasted against the NCBI nt. database to identify a possible source of the resistance genes.

Supplementary Table 5 | Set of 27 clinically relevant resistance genes
The table contains, in fasta format, the 27 clinically relevant resistance genes used in the mapping analysis of the deeply sequenced metagenome (Supplementary Fig. 4).

>ctx-m-15
ATGGTTAAAAAATCACTGCGCCAGTTCACGCTGATGGCGACGGCAACCGTCACGCTGTTGTTAGGAA
GTGTGCCGCTGTATGCGCAAACGGCGGACGTACAGCAAAAACTTGCCGAATTAGAGCGGCAGTCGGG
AGGCAGACTGGGTGTGGCATTGATTAACACAGCAGATAATTCGCAAATACTTTATCGTGCTGATGAG
CGCTTTGCGATGTGCAGCACCAGTAAAGTGATGGCCGCGGCCGCGGTGCTGAAGAAAAGTGAAAGCG
AACCGAATCTGTTAAATCAGCGAGTTGAGATCAAAAAATCTGACCTTGTTAACTATAATCCGATTGC
GGAAAAGCACGTCAATGGGACGATGTCACTGGCTGAGCTTAGCGCGGCCGCGCTACAGTACAGCGAT
AACGTGGCGATGAATAAGCTGATTGCTCACGTTGGCGGCCCGGCTAGCGTCACCGCGTTCGCCCGACA
GCTGGGAGACGAAACGTTCCGTCTCGACCGTACCGAGCCGACGTTAAACACCGCCATTCCGGGCGATC
CGCGTGATACCACTTCACCTCGGGCAATGGCGCAAACTCTGCGGAATCTGACGCTGGGTAAAGCATTG
GGCGACAGCCAACGGGCGCAGCTGGTGACATGGATGAAAGGCAATACCACCGGTGCAGCGAGCATTC
AGGCTGGACTGCCTGCTTCCTGGGTTGTGGGGGATAAAACCGGCAGCGGTGGCTATGGCACCACCAA
CGATATCGCGGTGATCTGGCCAAAAGATCGTGCGCCGCTGATTCTGGTCACTTACTTCACCCAGCCTC
AACCTAAGGCAGAAAGCCGTCGCGATGTATTAGCGTCGGCGGCTAAAATCGTCACCGACGGTTTGTA
A
>ctx-m-14
ATGGTGACAAAGAGAGTGCAACGGATGATGTTCGCGGCGGCGGCGTGCATTCCGCTGCTGCTGGGCA
GCGCGCCGCTTTATGCGCAGACGAGTGCGGTGCAGCAAAAGCTGGCGGCGCTGGAGAAAAGCAGCGG
AGGGCGGCTGGGCGTCGCGCTCATCGATACCGCAGATAATACGCAGGTGCTTTATCGCGGTGATGAA
CGCTTTCCAATGTGCAGTACCAGTAAAGTTATGGCGGCCGCGGCGGTGCTTAAGCAGAGTGAAACGC
AAAAGCAGCTGCTTAATCAGCCTGTCGAGATCAAGCCTGCCGATCTGGTTAACTACAATCCGATTGCC
GAAAAACACGTCAACGGCACAATGACGCTGGCAGAACTGAGCGCGGCCGCGTTGCAGTACAGCGACA
ATACCGCCATGAACAAATTGATTGCCCAGCTCGGTGGCCCGGGAGGCGTGACGGCTTTTGCCCGCGCG
ATCGGCGATGAGACGTTTCGTCTGGATCGCACTGAACCTACGCTGAATACCGCCATTCCCGGCGACCC
GAGAGACACCACCACGCCGCGGGCGATGGCGCAGACGTTGCGTCAGCTTACGCTGGGTCATGCGCTGG
GCGAAACCCAGCGGGCGCAGTTGGTGACGTGGCTCAAAGGCAATACGACCGGCGCAGCCAGCATTCG
GGCCGGCTTACCGACGTCGTGGACTGTGGGTGATAAGACCGGCAGCGGCGACTACGGCACCACCAATG
ATATTGCGGTGATCTGGCCGCAGGGTCGTGCGCCGCTGGTTCTGGTGACCTATTTTACCCAGCCGCAA
CAGAACGCAGAGAGCCGCCGCGATGTGCTGGCTTCAGCGGCGAGAATCATCGCCGAAGGGCTGTAA
>ctx-m-9
ATGGTGACAAAGAGAGTGCAACGGATGATGTTCGCGGCGGCGGCGTGCATTCCGCTGCTGCTGGGCA
GCGCGCCGCTTTATGCGCAGACGAGTGCGGTGCAGCAAAAGCTGGCGGCGCTGGAGAAAAGCAGCGG
AGGGCGGCTGGGCGTCGCGCTCATCGATACCGCAGATAATACGCAGGTGCTTTATCGCGGTGATGAA
CGCTTTCCAATGTGCAGTACCAGTAAAGTTATGGCGGCCGCGGCGGTGCTTAAGCAGAGTGAAACGC
AAAAGCAGCTGCTTAATCAGCCTGTCGAGATCAAGCCTGCCGATCTGGTTAACTACAATCCGATTGCC
GAAAAACACGTCAACGGCACAATGACGCTGGCAGAGCTGAGCGCGGCCGCGTTGCAGTACAGCGACA
ATACCGCCATGAACAAATTGATTGCCCAGCTCGGTGGCCCGGGAGGCGTGACGGCTTTTGCCCGCGCG
ATCGGCGATGAGACGTTTCGTCTGGATCGCACTGAACCTACGCTGAATACCGCCATTCCCGGCGACCC
GAGAGACACCACCACGCCGCGGGCGATGGCACAGACGTTGCGTCAGCTTACGCTGGGTCATGCGCTGG
GCGAAACCCAGCGGGCGCAGTTGGTGACGTGGCTCAAAGGCAATACGACCGGCGCAGCCAGCATTCG
GGCCGGCTTACCGACGTCGTGGACTGCAGGTGATAAGACCGGCAGCGGCGACTACGGCACCACCAATG
ATATTGCGGTGATCTGGCCGCAGGGTCGTGCGCCGCTGGTTCTGGTGACCTATTTTACCCAGCCGCAA
CAGAACGCAGAGAGCCGCCGCGATGTGCTGGCTTCAGCGGCGAGAATCATCGCCGAAGGGCTGTAA
>ctx-m-3

ATGGTTAAAAAATCACTGCGCCAGTTCACGCTGATGGCGACGGCAACCGTCACGCTGTTGTTAGGAA
GTGTGCCGCTGTATGCGCAAACGGCGGACGTACAGCAAAAACTTGCCGAATTAGAGCGGCAGTCGGG
AGGCAGACTGGGTGTGGCATTGATTAACACAGCAGATAATTCGCAAATACTTTATCGTGCTGATGAG
CGCTTTGCGATGTGCAGCACCAGTAAAGTGATGGCCGCGGCCGCGGTGCTGAAGAAAAGTGAAAGCG
AACCGAATCTGTTAAATCAGCGAGTTGAGATCAAAAAATCTGACCTTGTTAACTATAATCCGATTGC
GGAAAAGCACGTCAATGGGACGATGTCACTGGCTGAGCTTAGCGCGGCCGCGCTACAGTACAGCGAT
AACGTGGCGATGAATAAGCTGATTGCTCACGTTGGCGGCCCGGCTAGCGTCACCGCGTTCGCCCGACA
GCTGGGAGACGAAACGTTCCGTCTCGACCGTACCGAGCCGACGTTAAACACCGCCATTCCGGGCGATC
CGCGTGATACCACTTCACCTCGGGCAATGGCGCAAACTCTGCGGAATCTGACGCTGGGTAAAGCATTG
GGCGACAGCCAACGGGCGCAGCTGGTGACATGGATGAAAGGCAATACCACCGGTGCAGCGAGCATTC
AGGCTGGACTGCCTGCTTCCTGGGTTGTGGGGGATAAAACCGGCAGCGGTGACTATGGCACCACCAA
CGATATCGCGGTGATCTGGCCAAAAGATCGTGCGCCGCTGATTCTGGTCACTTACTTCACCCAGCCTC
AACCTAAGGCAGAAAGCCGTCGCGATGTATTAGCGTCGGCGGCTAAAATCGTCACCGACGGTTTGTA
A
>ctx-m-1
ATGGTTAAAAAATCACTGCGTCAGTTCACGCTGATGGCGACGGCAACCGTCACGCTGTTGTTAGGAA
GTGTGCCGCTGTATGCGCAAACGGCGGACGTACAGCAAAAACTTGCCGAATTAGAGCGGCAGTCGGG
AGGAAGACTGGGTGTGGCATTGATTAACACAGCAGATAATTCGCAAATACTTTATCGTGCTGATGAG
CGCTTTGCGATGTGCAGCACCAGTAAAGTGATGGCCGTGGCCGCGGTGCTGAAGAAAAGTGAAAGCG
AACCGAATCTGTTAAATCAGCGAGTTGAGATCAAAAAATCTGACTTGGTTAACTATAATCCGATTGC
GGAAAAGCACGTCGATGGGACGATGTCACTGGCTGAGCTTAGCGCGGCCGCGCTACAGTACAGCGAT
AACGTGGCGATGAATAAGCTGATTTCTCACGTTGGCGGCCCGGCTAGCGTCACCGCGTTCGCCCGACA
GCTGGGAGACGAAACGTTCCGTCTCGACCGTACCGAGCCGACGTTAAACACCGCCATTCCGGGCGATC
CGCGTGATACCACTTCACCTCGGGCAATGGCGCAAACTCTGCGTAATCTGACGCTGGGTAAAGCATTG
GGTGACAGCCAACGGGCGCAGCTGGTGACATGGATGAAAGGCAATACCACCGGTGCAGCGAGCATTC
AGGCTGGACTGCCTGCTTCCTGGGTTGTGGGGGATAAAACCGGCAGCGGTGACTATGGCACCACCAA
CGATATCGCGGTGATCTGGCCAAAAGATCGTGCGCCGCTGATTCTGGTCACTTACTTCACCCAGCCTC
AACCTAAGGCAGAAAGCCGTCGCGATGTATTAGCGTCGGCGGCTAAAATCGTCACCAACGGTTTGTA
A
>tem-1
ATGAGTATTCAACATTTCCGTGTCGCCCTTATTCCCTTTTTTGCGGCATTTTGCCTTCCTGTTTTTGC
TCACCCAGAAACGCTGGTGAAAGTAAAAGATGCTGAAGATCAGTTGGGTGCACGAGTGGGTTACATC
GAACTGGATCTCAACAGCGGTAAGATCCTTGAGAGTTTTCGCCCCGAAGAACGTTTTCCAATGATGA
GCACTTTTAAAGTTCTGCTATGTGGCGCGGTATTATCCCGTGTTGACGCCGGGCAAGAGCAACTCGGT
CGCCGCATACACTATTCTCAGAATGACTTGGTTGAGTACTCACCAGTCACAGAAAAGCATCTTACGG
ATGGCATGACAGTAAGAGAATTATGCAGTGCTGCCATAACCATGAGTGATAACACTGCGGCCAACTT
ACTTCTGACAACGATCGGAGGACCGAAGGAGCTAACCGCTTTTTTGCACAACATGGGGGATCATGTA
ACTCGCCTTGATCGTTGGGAACCGGAGCTGAATGAAGCCATACCAAACGACGAGCGTGACACCACGA
TGCCTGCAGCAATGGCAACAACGTTGCGCAAACTATTAACTGGCGAACTACTTACTCTAGCTTCCCGG
CAACAATTAATAGACTGGATGGAGGCGGATAAAGTTGCAGGACCACTTCTGCGCTCGGCCCTTCCGG
CTGGCTGGTTTATTGCTGATAAATCTGGAGCCGGTGAGCGTGGGTCTCGCGGTATCATTGCAGCACT
GGGGCCAGATGGTAAGCCCTCCCGTATCGTAGTTATCTACACGACGGGGAGTCAGGCAACTATGGAT
GAACGAAATAGACAGATCGCTGAGATAGGTGCCTCACTGATTAAGCATTGGTAA
>tetA
ATGAAATCTAACAATGCGCTCATCGTCATCCTCGGCACCGTCACCCTGGATGCTGTAGGCATAGGCTT
GGTTATGCCGGTACTGCCGGGCCTCTTGCGGGATATCGTCCATTCCGACAGCATCGCCAGTCACTATG
GCGTGCTGCTAGCGCTATATGCGTTGATGCAATTTCTATGCGCACCCGTTCTCGGAGCACTGTCCGAC
CGCTTTGGCCGCCGCCCAGTCCTGCTCGCTTCGCTACTTGGAGCCACTATCGACTACGCGATCATGGC
GACCACACCCGTCCTGTGGATCCTCTACGCCGGACGCATCGTGGCCGGCATCACCGGCGCCACAGGTG

CGGTTGCTGGCGCCTATATCGCCGACATCACCGATGGGGAAGATCGGGCTCGCCACTTCGGGCTCATG
AGCGCTTGTTTCGGCGTGGGTATGGTGGCAGGCCCCGTGGCCGGGGGACTGTTGGGCGCCATCTCCTT
GCATGCACCATTCCTTGCGGCGGCGGTGCTCAACGGCCTCAACCTACTACTGGGCTGCTTCCTAATGC
AGGAGTCGCATAAGGGAGAGCGTCGACCGATGCCCTTGAGAGCCTTCAACCCAGTCAGCTCCTTCCGG
TGGGCGCGGGGCATGACTATCGTCGCCGCACTTATGACTGTCTTCTTTATCATGCAACTCGTAGGACA
GGTGCCGGCAGCGCTCTGGGTCATTTTCGGCGAGGACCGCTTTCGCTGGAGCGCGACGATGATCGGCC
TGTCGCTTGCGGTATTCGGAATCTTGCACGCCCTCGCTCAAGCCTTCGTCACTGGTCCCGCCACCAAA
CGTTTCGGCGAGAAGCAGGCCATTATCGCCGGCATGGCGGCCGACGCGCTGGGCTACGTCTTGCTGGC
GTTCGCGACGCGAGGCTGGATGGCCTTCCCCATTATGATTCTTCTCGCTTCCGGCGGCATCGGGATGC
CCGCGTTGCAGGCCATGCTGTCCAGGCAGGTAGATGACGACCATCAGGGACAGCTTCAAGGATCGCTC
GCGGCTCTTACCAGCCTAACTTCGATCACTGGACCGCTGATCGTCACGGCGATTTATGCCGCCTCGGC
GAGCACATGGAACGGGTTGGCATGGATTGTAGGCGCCGCCCTATACCTTGTCTGCCTCCCCGCGTTGC
GTCGCGGTGCATGGAGCCGGGCCACCTCGACCTGA
>tetM
ATGAAAATTATTAATATTGGAGTTTTAGCTCATGTTGATGCAGGAAAAACTACCTTAACAGAAAGCT
TATTATATAACAGTGGAGCGATTACAGAATTAGGAAGCGTGGACAAAGGTACAACGAGGACGGATA
ATACGCTTTTAGAACGTCAGAGAGGAATTACAATTCAGACAGGAATAACCTCTTTTCAGTGGGAAAA
TACGAAGGTGAACATCATAGACACGCCAGGACATATGGATTTCTTAGCAGAAGTATATCGTTCATTA
TCAGTTTTAGATGGGGCAATTCTACTGATTTCTGCAAAAGATGGCGTACAAGCACAAACTCGTATAT
TATTTCATGCACTTAGGAAAATGGGGATTCCCACAATCTTTTTTATCAATAAGATTGACCAAAATGG
AATTGATTTATCAACGGTTTATCAGGATATTAAAGAGAAACTTTCTGACGAAATTGTAATCAAACAG
AAGGTAGAACTGTATCCTAATATGTGTGTGACGAACTTTACCGAATCTGAACAATGGGATACGGTAA
TAGAGGGAAACGATGACCTTTTAGAGAAATATATGTCCGGTAAATCATTAGAAGCATTGGAACTCGA
ACAAGAGGAAAGCATAAGATTTCATAATTGTTCCCTGTTCCCTGTTTATCACGGAAGTGCAAAAAAC
AATATAGGGATTGATAACCTTATAGAAGTGATTACGAATAAATTTTATTCATCAACACATCGAGGTC
CGTCTGAACTTTGCGGAAATGTTTTCAAAATTGAATATACAAAAAAAAGACAACGTCTTGCATATAT
ACGCCTTTATAGTGGAGTACTACATTTACGAGATTCGGTTAGAGTATCAGAAAAAGAAAAAATAAA
AGTTACAGAAATGTATACTTCAATAAATGGTGAATTATGTAAGATTGATAGAGCTTATTCTGGAGA
AATTGTTATTTTGCAAAATGAGTTTTTGAAGTTAAATAGTGTTCTTGGAGATACAAAACTATTGCCA
CAGAGAAAAAAGATTGAAAATCCGCACCCTCTACTACAAACAACTGTTGAACCGAGTAAACCTGAAC
AGAGAGAAATGTTGCTTGATGCCCTTTTGGAAATCTCAGATAGTGATCCGCTTCTACGATATTACGT
GGATTCTACGACACATGAAATTATACTTTCTTTCTTAGGGAAAGTACAAATGGAAGTGATTAGTGCA
CTGTTGCAAGAAAAGTATCATGTGGAGATAGAACTAAAAGAGCCTACAGTCATTTATATGGAGAGAC
CGTTAAAAAATGCAGAATATACCATTCACATCGAAGTGCCGCCAAATCCTTTCTGGGCTTCCATTGG
TTTATCTGTATCACCGCTTCCGTTGGGAAGTGGAATGCAGTATGAGAGCTCGGTTTCTCTTGGATAC
TTAAATCAATCATTTCAAAATGCAGTTATGGAAGGGATACGCTATGGTTGCGAACAAGGATTATATG
GTTGGAATGTGACGGATTGTAAAATCTGTTTTAAGTATGGCTTATACTATAGCCCTGTTAGTACCCC
AGCAGATTTTCGGATGCTTGCTCCTATTGTATTGGAACAAGTCTTAAAAAAAGCTGGAACAGAATTG
TTAGAGCCATATCTTAGTTTTAAAATTTATGCGCCACAGGAATATCTTTCACGAGCATACAACGATG
CTCCTAAATATTGTGCGAACATCGTAGACACTCAATTGAAAAATAATGAGGTCATTCTTAGTGGAGA
AATCCCTGCTCGGTGTATTCAAGAATATCGTAGTGATTTAACTTTCTTTACAAATGGACGTAGTGTT
TGTTTAACAGAGTTAAAAGGGTACCATGTTACTACCGGTGAACCTGTTTGCCAGCCCCGTCGTCCAA
ATAGTCGGATAGATAAAGTACGATATATGTTCAATAAAATAACTTAG
>tetK
ATGGTTTTAAATGTTTCTTTACCTGATATTGCAAATCATTTTAATACTACTCCTGGAATTACAAACT
GGGTAAACACTGCATATATGTTAACTTTTTCGATAGGAACAGCAGTATATGGAAAATTATCTGATTA
TATAAATATAAAAAAATTGTTAATTATTGGTATTAGTTTGAGCTGTCTTGGTTCATTGATTGCTTTT
ATTGGTCACAATCACTTTTTTATTTTGATTTTTGGTAGGTTAGTACAAGGAGTAGGATCTGCTGCAT
TCCCTTCACTGATTATGGTGGTTGTAGCTAGAAATATTACAAGAAAAAAACAAGGCAAAGCCTTTGG

TTTTATAGGATCAATTGTAGCTTTAGGTGAAGGGTTAGGTCCTTCAATAGGGGGAATAATAGCACAT
TATATTCATTGGTCTTACCTACTTATACTTCCTATGATTACAATAGTAACTATACCTTTTCTTATTA
AAGTAATGGTACCTGGTAAATCAACAAAAAATACATTAGATATCGTAGGTATTGTTTTAATGTCTAT
AAGTATTATATGTTTTATGTTATTTACGACAAATTATAATTGGACTTTTTTAATACTCTTCACAATC
TTTTTTGTGATTTTTATTAAACATATTTCAAGAGTTTCTAACCCTTTTATTAATCCTAAACTAGGGA
AAAACATTCCGTTTATGCTTGGTTTGTTTTCTGGTGGGCTAATATTTTCTATAGTAGCTGGTTTTAT
ATCAATGGTGCCTTATATGATGAAAACTATTTATCATGTAAATGTAGCGACAATAGGTAATAGTGTT
ATTTTTCCTGGAACCATGAGTGTTATTGTTTTTGGTTATTTTGGTGGTTTTTTAGTGGATAGAAAAG
GATCATTATTTGTTTTTATTTTAGGATCATTGTCTATCTCTATAAGTTTTTTAACTATTGCATTTTT
TGTTGAGTTTAGTATGTGGTTGACTACTTTTATGTTTATATTTGTTATGGGCGGATTATCTTTTACT
AAAACAGTTATATCAAAAATAGTATCAAGTAGTCTTTCTGAAGAAGAAGTTGCTTCTGGAATGAGTT
TGCTAAATTTCACAAGTTTTTTATCAGAGGGAACAGGTATAGCAATTGTAGGAGGTTTATTGTCACT
ACAATTGATTAATCGTAAACTAGTTCTGGAATTTATAAATTATTCTTCTGGAGTGTATAGTAATATT
CTTGTAGCCATGGCTATCCTTATTATTTTATGTTGTCTTTTGACGATTATTGTATTTAAACGTTCTG
AAAAGCAGTTTGAATAG
>tetO
ATGAAAATAATTAACTTAGGCATTCTGGCTCACGTTGACGCAGGAAAGACAACATTAACGGAAAGTT
TATTGTATACCAGTGGTGCAATTGCAGAACTAGGGAGCGTAGATGAAGGCACAACAAGGACAGATAC
AATGAATTTGGAGCGTCAAAGGGGAATCACTATCCAGACAGCAGTGACATCTTTTCAGTGGGAGGAT
GTAAAAGTCAACATTATAGATACGCCAGGCCATATGGATTTTTTGGCGGAAGTATACCGTTCTTTAT
CCGTATTAGACGGAGCAGTATTATTAGTTTCTGCAAAGGATGGCATACAGGCACAGACCCGTATACT
GTTTCATGCACTACAGATAATGAAGATTCCGACAATTTTTTTCATCAATAAAATTGACCAAGAGGGG
ATTGATTTGCCAATGGTATATCGGGAAATGAAAGCAAAGCTTTCTTCGGAAATTATAGTGAAGCAAA
AGGTTGGGCAGCATCCCCATATAAATGTAACGGACAATGACGATATGGAACAGTGGGATGCGGTAAT
TATGGGAAACGATGAACTATTAGAGAAATATATGTCAGGGAAACCGTTTAAAATGTCAGAACTGGA
ACAGGAAGAAAACAGGAGATTCCAAAACGGAACGTTATTTCCCGTTTATCACGGAAGCGCTAAAAAC
AATCTGGGGACTCGGCAGCTTATAGAAGTAATTGCCAGTAAATTTTATTCATCAACGCCTGAAGGTC
AATCTGAACTATGCGGGCAGGTTTTTAAGATTGAATATTCAGAGAAAAGGCGGCGTTTTGTTTATGT
GCGTATATATAGCGGAACATTGCATTTGAGGGATGTTATTAGAATATCTGAAAAAGAGAAAATAAA
AATCACAGAGATGTATGTTCCGACAAACGGTGAATTATATTCATCCGATACAGCCTGCTCTGGTGAT
ATTGTAATTTTACCAAATGATGTTTTGCAGCTAAACAGTATTTTGGGGAACGAAATACTGTTGCCGC
AGAGAAAATTTATTGAAAATCCTCTCCCTATGATCCAAACAACGATTGCAGTAAAGAAATCTGAACA
GCGGGAAATATTGCTTGGGGCACTTACAGAAATTTCAGATTGCGACCCTCTTTTAAAATATTATGTG
GATACTACAACGCATGAGATTATACTTTCTTTTTTGGGGAATGTGCAGATGGAAGTCATTTGTGCCA
TCCTTGAGGAAAAATATCATGTGGAGGCAGAAATAAAAGAGCCTACTGTTATATATATGGAAAGACC
GCTTAGAAAAGCAGAATATACCATCCACATAGAAGTCCCGCCAAATCCTTTCTGGGCTTCTGTCGGG
TTGTCCATAGAGCCGCTCCCTATTGGAAGCGGAGTGCAGTATGAAAGCAGAGTTTCACTTGGATATT
TAAATCAATCGTTCCAAAATGCGGTTATGGAGGGGGTTCTTTATGGCTGCGAGCAGGGGCTGTATGG
ATGGAAAGTGACAGACTGTAAAATCTGTTTTGAATATGGATTGTATTATAGTCCTGTAAGTACCCCC
GCAGACTTTCGGCTGCTTTCCCCTATCGTATTGGAGCAGGCTTTAAAAAAAGCAGGGACAGAACTAT
TAGAGCCATATCTCCACTTTGAAATTTATGCACCGCAGGAATATCTCTCACGGGCGTATCATGATGCT
CCAAGGTATTGTGCAGATATTGTAAGTACTCAGATAAAGAATGACGAGGTCATTCTGAAAGGAGAAA
TCCCTGCTAGATGTATTCAAGAATACAGGAACGATTTAACTTATTTCACAAATGGGCAGGGAGTCTG
CTTGACAGAGTTAAAAGGATACCAGCCAGCTATTGGTAAATTTATTTGCCAACCCCGCCGCCCGAAT
AGCCGTATAGATAAGGTTCGGCATATGTTCCACAAGTTAGCTTAA
>ndm-1
AAAGGAAAACTTGATGGAATTGCCCAATATTATGCACCCGGTCGCGAAGCTGAGCACCGCATTAGCC
GCTGCATTGATGCTGAGCGGGTGCATGCCCGGTGAAATCCGCCCGACGATTGGCCAGCAAATGGAAA
CTGGCGACCAACGGTTTGGCGATCTGGTTTTCCGCCAGCTCGCACCGAATGTCTGGCAGCACACTTCC

TATCTCGACATGCCGGGTTTCGGGGCAGTCGCTTCCAACGGTTTGATCGTCAGGGATGGCGGCCGCGT
GCTGGTGGTCGATACCGCCTGGACCGATGACCAGACCGCCCAGATCCTCAACTGGATCAAGCAGGAGA
TCAACCTGCCGGTCGCGCTGGCGGTGGTGACTCACGCGCATCAGGACAAGATGGGCGGTATGGACGCG
CTGCATGCGGCGGGGATTGCGACTTATGCCAATGCGTTGTCGAACCAGCTTGCCCCGCAAGAGGGGAT
GGTTGCGGCGCAACACAGCCTGACTTTCGCCGCCAATGGCTGGGTCGAACCAGCAACCGCGCCCAACT
TTGGCCCGCTCAAGGTATTTTACCCCGGCCCCGGCCACACCAGTGACAATATCACCGTTGGGATCGAC
GGCACCGACATCGCTTTTGGTGGCTGCCTGATCAAGGACAGCAAGGCCAAGTCGCTCGGCAATCTCGG
TGATGCCGACACTGAGCACTACGCCGCGTCAGCGCGCGCGTTTGGTGCGGCGTTCCCCAAGGCCAGCA
TGATCGTGATGAGCCATTCCGCCCCCGATAGCCGCGCCGCAATCACTCATACGGCCCGCATGGCCGAC
AAGCTGCGCTGA
>kpc-2
ATGTCACTGTATCGCCGTCTAGTTCTGCTGTCTTGTCTCTCATGGCCGCTGGCTGGCTTTTCTGCCAC
CGCGCTGACCAACCTCGTCGCGGAACCATTCGCTAAACTCGAACAGGACTTTGGCGGCTCCATCGGTG
TGTACGCGATGGATACCGGCTCAGGCGCAACTGTAAGTTACCGCGCTGAGGAGCGCTTCCCACTGTGC
AGCTCATTCAAGGGCTTTCTTGCTGCCGCTGTGCTGGCTCGCAGCCAGCAGCAGGCCGGCTTGCTGGA
CACACCCATCCGTTACGGCAAAAATGCGCTGGTTCCGTGGTCACCCATCTCGGAAAAATATCTGACAA
CAGGCATGACGGTGGCGGAGCTGTCCGCGGCCGCCGTGCAATACAGTGATAACGCCGCCGCCAATTTG
TTGCTGAAGGAGTTGGGCGGCCCGGCCGGGCTGACGGCCTTCATGCGCTCTATCGGCGATACCACGTT
CCGTCTGGACCGCTGGGAGCTGGAGCTGAACTCCGCCATCCCAGGCGATGCGCGCGATACCTCATCGC
CGCGCGCCGTGACGGAAAGCTTACAAAAACTGACACTGGGCTCTGCACTGGCTGCGCCGCAGCGGCAG
CAGTTTGTTGATTGGCTAAAGGGAAACACGACCGGCAACCACCGCATCCGCGCGGCGGTGCCGGCAG
ACTGGGCAGTCGGAGACAAAACCGGAACCTGCGGAGTGTATGGCACGGCAAATGACTATGCCGTCGT
CTGGCCCACTGGGCGCGCACCTATTGTGTTGGCCGTCTACACCCGGGCGCCTAACAAGGATGACAAGC
ACAGCGAGGCCGTCATCGCCGCTGCGGCTAGACTCGCGCTCGAGGGATTGGGCGTCAACGGGCAGTAA
>kpc-3
ATGTCACTGTATCGCCGTCTAGTTCTGCTGTCTTGTCTCTCATGGCCGCTGGCTGGCTTTTCTGCCAC
CGCGCTGACCAACCTCGTCGCGGAACCATTCGCTAAACTCGAACAGGACTTTGGCGGCTCCATCGGTG
TGTACGCGATGGATACCGGCTCAGGCGCAACTGTAAGTTACCGCGCTGAGGAGCGCTTCCCACTGTGC
AGCTCATTCAAGGGCTTTCTTGCTGCCGCTGTGCTGGCTCGCAGCCAGCAGCAGGCCGGCTTGCTGGA
CACACCCATCCGTTACGGCAAAAATGCGCTGGTTCCGTGGTCACCCATCTCGGAAAAATATCTGACAA
CAGGCATGACGGTGGCGGAGCTGTCCGCGGCCGCCGTGCAATACAGTGATAACGCCGCCGCCAATTTG
TTGCTGAAGGAGTTGGGCGGCCCGGCCGGGCTGACGGCCTTCATGCGCTCTATCGGCGATACCACGTT
CCGTCTGGACCGCTGGGAGCTGGAGCTGAACTCCGCCATCCCAGGCGATGCGCGCGATACCTCATCGC
CGCGCGCCGTGACGGAAAGCTTACAAAAACTGACACTGGGCTCTGCACTGGCTGCGCCGCAGCGGCAG
CAGTTTGTTGATTGGCTAAAGGGAAACACGACCGGCAACCACCGCATCCGCGCGGCGGTGCCGGCAG
ACTGGGCAGTCGGAGACAAAACCGGAACCTGCGGAGTGTATGGCACGGCAAATGACTATGCCGTCGT
CTGGCCCACTGGGCGCGCACCTATTGTGTTGGCCGTCTACACCCGGGCGCCTAACAAGGATGACAAGT
ACAGCGAGGCCGTCATCGCCGCTGCGGCTAGACTCGCGCTCGAGGGATTGGGCGTCAACGGGCAGTAA
>shv-1
ATGCGTTATATTCGCCTGTGTATTATCTCCCTGTTAGCCACCCTGCCGCTGGCGGTACACGCCAGCCC
GCAGCCGCTTGAGCAAATTAAACTAAGCGAAAGCCAGCTGTCGGGCCGCGTAGGCATGATAGAAATG
GATCTGGCCAGCGGCCGCACGCTGACCGCCTGGCGCGCCGATGAACGCTTTCCCATGATGAGCACCTT
TAAAGTAGTGCTCTGCGGCGCAGTGCTGGCGCGGGTGGATGCCGGTGACGAACAGCTGGAGCGAAAG
ATCCACTATCGCCAGCAGGATCTGGTGGACTACTCGCCGGTCAGCGAAAAACACCTTGCCGACGGCAT
GACGGTCGGCGAACTCTGCGCCGCCGCCATTACCATGAGCGATAACAGCGCCGCCAATCTGCTACTGG
CCACCGTCGGCGGCCCCGCAGGATTGACTGCCTTTTTGCGCCAGATCGGCGACAACGTCACCCGCCTT
GACCGCTGGGAAACGGAACTGAATGAGGCGCTTCCCGGCGACGCCCGCGACACCACTACCCCGGCCAG
CATGGCCGCGACCCTGCGCAAGCTGCTGACCAGCCAGCGTCTGAGCGCCCGTTCGCAACGGCAGCTGC
TGCAGTGGATGGTGGACGATCGGGTCGCCGGACCGTTGATCCGCTCCGTGCTGCCGGCGGGCTGGTTT

ATCGCCGATAAGACCGGAGCTGGCGAGCGGGGTGCGCGCGGGATTGTCGCCCTGCTTGGCCCGAATAA
CAAAGCAGAGCGCATTGTGGTGATTTATCTGCGGGATACCCCGGCGAGCATGGCCGAGCGAAATCAG
CAAATCGCCGGGATCGGCGCGGCGCTGATCGAGCACTGGCAACGCTAAA
>oxa-1
ATGAAAAACACAATACATATCAACTTCGCTATTTTTTTAATAATTGCAAATATTATCTACAGCAGCG
CCAGTGCATCAACAGATATCTCTACTGTTGCATCTCCATTATTTGAAGGAACTGAAGGTTGTTTTTT
ACTTTACGATGCATCCACAAACGCTGAAATTGCTCAATTCAATAAAGCAAAGTGTGCAACGCAAATG
GCACCAGATTCAACTTTCAAGATCGCATTATCACTTATGGCATTTGATGCGGAAATAATAGATCAGA
AAACCATATTCAAATGGGATAAAACCCCCAAAGGAATGGAGATCTGGAACAGCAATCATACACCAAA
GACGTGGATGCAATTTTCTGTTGTTTGGGTTTCGCAAGAAATAACCCAAAAAATTGGATTAAATAAA
ATCAAGAATTATCTCAAAGATTTTGATTATGGAAATCAAGACTTCTCTGGAGATAAAGAAAGAAACA
ACGGATTAACAGAAGCATGGCTCGAAAGTAGCTTAAAAATTTCACCAGAAGAACAAATTCAATTCCT
GCGTAAAATTATTAATCACAATCTCCCAGTTAAAAACTCAGCCATAGAAAACACCATAGAGAACATG
TATCTACAAGATCTGGATAATAGTACAAAACTGTATGGGAAAACTGGTGCAGGATTCACAGCAAATA
GAACCTTACAAAACGGATGGTTTGAAGGGTTTATTATAAGCAAATCAGGACATAAATATGTTTTTGT
GTCCGCACTTACAGGAAACTTGGGGTCGAATTTAACATCAAGCATAAAAGCCAAGAAAAATGCGATC
ACCATTCTAAACACACTAAATTTATAA
>vim-2
ATGTTCAAACTTTTGAGTAAGTTATTGGTCTATTTGACCGCGTCTATCATGGCTATTGCGAGTCCGC
TCGCTTTTTCCGTAGATTCTAGCGGTGAGTATCCGACAGTCAGCGAAATTCCGGTCGGGGAGGTCCG
GCTTTACCAGATTGCCGATGGTGTTTGGTCGCATATCGCAACGCAGTCGTTTGATGGCGCAGTCTACC
CGTCCAATGGTCTCATTGTCCGTGATGGTGATGAGTTGCTTTTGATTGATACAGCGTGGGGTGCGAA
AAACACAGCGGCACTTCTCGCGGAGATTGAGAAGCAAATTGGACTTCCTGTAACGCGTGCAGTCTCC
ACGCACTTTCATGACGACCGCGTCGGCGGCGTTGATGTCCTTCGGGCGGCTGGGGTGGCAACGTACGC
ATCACCGTCGACACGCCGGCTAGCCGAGGTAGAGGGGAACGAGATTCCCACGCACTCTCTAGAAGGAC
TCTCATCGAGCGGGGACGCAGTGCGCTTCGGTCCAGTAGAACTCTTCTATCCTGGTGCTGCGCATTCG
ACCGACAACTTAGTTGTGTACGTCCCGTCTGCGAGTGTGCTCTATGGTGGTTGTGCGATTTATGAGT
TGTCACGCACGTCTGCGGGGAACGTGGCCGATGCCGATCTGGCTGAATGGCCCACCTCCATTGAGCGG
ATTCAACAACACTACCCGGAAGCACAGTTCGTCATTCCGGGGCACGGCCTGCCGGGCGGTCTAGACTT
GCTCAAGCACACAACGAATGTTGTAAAAGCGCACACAAATCGCTCAGTCGTTGAGTAG
>vanA
ATGAATAGAATAAAAGTTGCAATACTGTTTGGGGGTTGCTCAGAGGAGCATGACGTATCGGTAAAAT
CTGCAATAGAGATAGCCGCTAACATTAATAAAGAAAAATACGAGCCGTTATACATTGGAATTACGAA
ATCTGGTGTATGGAAAATGTGCGAAAAACCTTGCGCGGAATGGGAAAACGACAATTGCTATTCAGCT
GTACTCTCGCCGGATAAAAAAATGCACGGATTACTTGTTAAAAAGAACCATGAATATGAAATCAACC
ATGTTGATGTAGCATTTTCAGCTTTGCATGGCAAGTCAGGTGAAGATGGATCCATACAAGGTCTGTT
TGAATTGTCCGGTATCCCTTTTGTAGGCTGCGATATTCAAAGCTCAGCAATTTGTATGGACAAATCG
TTGACATACATCGTTGCGAAAAATGCTGGGATAGCTACTCCCGCCTTTTGGGTTATTAATAAAGATG
ATAGGCCGGTGGCAGCTACGTTTACCTATCCTGTTTTTGTTAAGCCGGCGCGTTCAGGCTCATCCTTC
GGTGTGAAAAAAGTCAATAGCGCGGACGAATTGGACTACGCAATTGAATCGGCAAGACAATATGACA
GCAAAATCTTAATTGAGCAGGCTGTTTCGGGCTGTGAGGTCGGTTGTGCGGTATTGGGAAACAGTGC
CGCGTTAGCTGTTGGCGAGGTGGACCAAATCAGGCTGCAGTACGGAATCTTTCGTATTCATCAGGAA
GTCGAGCCGGAAAAAGGCTCTGAAAACGCAGTTATAACCGTTCCCGCAGACCTTTCAGCAGAGGAGC
GAGGACGGATACAGGAAACGGCAAAAAAATATATAAAGCGCTCGGCTGTAGAGGTCTAG
>vanB
ATGAATAGAATAAAAGTCGCAATCATCTTCGGCGGTTGCTCGGAGGAACATGATGTGTCGGTAAAAT
CCGCAATAGAAATTGCTGCGAACATTAATACTGAAAAATTCGATCCGCACTACATCGGAATTACAAA
AAACGGCGTATGGAAGCTATGCAAGAAGCCATGTACGGAATGGGAAGCCGACAGTCTCCCCGCCATA
CTCTCCCCGGATAGGAAAACGCATGGGCTGCTTGTCATGAAAGAAAGCGAATACGAAACACGGCGTA

TTGATGTGGCTTTCCCGGTTTTGCATGGCAAATGCGGGGAGGATGGTGCGATACAGGGTCTGTTTGA
ATTGTCTGGTATCCCCTATGTAGGCTGCGATATTCAAAGCTCCGCAGCTTGCATGGACAAATCACTGG
CCTACATTCTTACAAAAAATGCGGGCATCGCCGTTCCCGAATTTCAAATGATTGATAAAGGTGACAA
GCCGGAGGCGGGTGCGCTTACCTACCCTGTCTTTGTGAAGCCGGCACGGTCAGGTTCGTCCTTTGGCG
TAACCAAAGTAAACGGTACGGAAGAACTTAACGCTGCGATAGAAGCGGCAGGACAATATGATGGAAA
AATCTTAATTGAGCAAGCGATTTCGGGCTGTGAGGTCGGGTGTGCGGTCATGGGGAACGAGGATGAT
TTGATTGTCGGCGAAGTGGATCAAATCCGGCTGAGCCACGGTATCTTCCGCATCCATCAGGAAAACG
AGCCGGAAAAAGGCTCAGAAAATGCGATGATTACAGTTCCCGCAGACATTCCGGTCGAGGAACGAAA
TCGGGTGCAGGAAACGGCAAAGAAAGTATATCGGGTGCTTGGATGCAGAGGGCTTGCCCGTGTTGAT
CTTTTTTTGCAGGAGGATGGCGGCATCGTTCTAAATGAGGTCAATACCCTGCCTGGTTTTACATCGT
ACAGCCGCTACCCACGTATGGTGGCCGCCGCAGGAATCACGCTTCCTGCACTGATTGACAGCCTGATT
ACATTGGCGTTAAAGAGGTGA
>catA
ATGGAGAAAAAAATCACTGGATATACCACCGTTGATATATCCCAATGGCATCGTAAAGAACATTTTG
AGGCATTTCAGTCAGTTGCTCAATGTACCTATAACCAGACCGTTCAGCTGGATATTACGGCCTTTTTA
AAGACCGTAAAGAAAAATAAGCACAAGTTTTATCCGGCCTTTATTCACATTCTTGCCCGCCTGATGA
ATGCTCATCCGGAATTCCGTATGGCAATGAAAGACGGTGAGCTGGTGATATGGGATAGTGTTCACCC
TTGTTACACCGTTTTCCATGAGCAAACTGAAACGTTTTCATCGCTCTGGAGTGAATACCACGACGAT
TTCCGGCAGTTTCTACACATATATTCGCAAGATGTGGCGTGTTACGGTGAAAACCTGGCCTATTTCCC
TAAAGGGTTTATTGAGAATATGTTTTTCGTCTCAGCCAATCCCTGGGTGAGTTTCACCAGTTTTGAT
TTAAACGTGGCCAATATGGACAACTTCTTCGCCCCCGTTTTCACCATGGGCAAATATTATACGCAAGG
CGACAAGGTGCTGATGCCGCTGGCGATTCAGGTTCATCATGCCGTTTGTGATGGCTTCCATGTCGGCA
GAATGCTTAATGAATTACAACAGTACTGCGATGAGTGGCAGGGCGGGGCGTA
>cfr
ATGAATTTTAATAATAAAACAAAGTATGGTAAAATACAGGAATTTTTAAGAAGTAATAATGAGCCT
GATTATAGAATAAAACAAATAACCAATGCGATTTTTAAACAAAGAATTAGTCGATTTGAGGATATG
AAGGTTCTTCCAAAATTACTTAGGGAGGATTTAATAAATAATTTTGGAGAAACAGTTTTGAATATCA
AGCTCTTAGCAGAGCAAAATTCAGAGCAAGTTACGAAAGTGCTTTTTGAAGTATCAAAGAATGAGAG
AGTAGAAACGGTAAACATGAAGTATAAAGCAGGTTGGGAGTCATTTTGTATATCATCACAATGCGGA
TGTAATTTTGGGTGTAAATTTTGTGCTACAGGCGACATTGGATTGAAAAAAAACCTAACTGTAGATG
AGATAACAGATCAAGTTTTATACTTCCATTTATTAGGTCATCAAATTGATAGCATTTCTTTTATGGG
AATGGGTGAAGCTCTAGCCAACCGTCAAGTATTTGATGCTCTTGATTCGTTTACGGATCCTAATTTA
TTTGCATTAAGTCCTCGTAGACTTTCTATATCAACGATTGGTATTATACCTAGTATCAAAAAAATAA
CCCAGGAATATCCTCAAGTAAATCTTACATTTTCATTACACTCACCTTATAGTGAGGAACGCAGCAA
ATTGATGCCAATAAATGATAGATACCCAATAGATGAGGTAATGAATATACTCGATGAACATATAAG
ATTAACTTCAAGGAAAGTATATATAGCTTATATCATGTTGCCTGGTGTAAATGATTCTCTTGAGCAT
GCAAACGAAGTTGTTAGCCTTCTTAAAAGTCGCTATAAATCAGGGAAGTTATATCATGTAAATTTGA
TACGATACAATCCTACAATAAGTGCACCTGAGATGTATGGAGAAGCAAACGAAGGGCAGGTAGAAGC
CTTTTACAAAGTTTTGAAGTCTGCTGGTATCCATGTCACAATTAGAAGTCAATTTGGGATTGATATT
GACGCTGCTTGTGGTCAATTATATGGTAATTATCAAAATAGCCAATAG
>ermA
ATGAACCAGAAAAACCCTAAAGACACGCAAAATTTTATTACTTCTAAAAAGCATGTAAAAGAAATAT
TGAATCACACGAATATCAGTAAACAAGACAACGTAATAGAAATCGGATCAGGAAAAGGACATTTTAC
CAAAGAGCTAGTCAAAATGAGTCGATCAGTTACTGCTATAGAAATTGATGGAGGCTTATGTCAAGTG
ACTAAAGAAGCGGTAAACCCCTCTGAGAATATAAAAGTGATTCAAACGGATATTCTAAAATTTTCCT
TCCCAAAACATATAAACTATAAGATATATGGTAATATTCCTTATAACATCAGTACGGATATTGTCAA
AAGAATTACCTTTGAAAGTCAGGCTAAATATAGCTATCTTATCGTTGAGAAGGGATTTGCGAAAAGA
TTGCAAAATCTGCAACGAGCTTTGGGTTTACTATTAATGGTGGAGATGGATATAAAAATGCTCAAAA
AAGTACCACCACTATATTTTCATCCTAAGCCAAGTGTAGACTCTGTATTGATTGTTCTTGAACGACA

TCAACCATTGATTTCAAAGAAGGACTACAAAAAGTATCGATCTTTTGTTTATAAGTGGGTAAACCGT
GAATATCGTGTTCTTTTCACTAAAAACCAATTCCGACAGGCTTTGAAGCATGCAAATGTCACTAATA
TTAATAAACTATCGAAGGAACAATTTCTTTCTATTTTCAATAGTTACAAATTGTTTCACTAA
>ermB
ATGAACAAAAATATAAAATATTCTCAAAACTTTTTAACGAGTGAAAAAGTACTCAACCAAATAATA
AAACAATTGAATTTAAAAGAAACCGATACCGTTTACGAAATTGGAACAGGTAAAGGGCATTTAACG
ACGAAACTGGCTAAAATAAGTAAACAGGTAACGTCTATTGAATTAGACAGTCATCTATTCAACTTAT
CGTCAGAAAAATTAAAACTGAACATTCGTGTCACTTTAATTCACCAAGATATTCTACAGTTTCAATT
CCCTAACAAACAGAGGTATAAAATTGTTGGGAGTATTCCTTACCATTTAAGCACACAAATTATTAAA
AAAGTGGTTTTTGAAAGCCATGCGTCTGACATCTATCTGATTGTTGAAGAAGGATTCTACAAGCGTA
CCTTGGATATTCACCGAACACTAGGGTTGCTCTTGCACACTCAAGTCTCGATTCAGCAATTGCTTAAG
CTGCCAGCGGAATGCTTTCATCCTAAACCAAAAGTAAACAGTGTCTTAATAAAACTTACCCGCCATA
CCACAGATGTTCCAGATAAATATTGGAAGCTATATACGTACTTTGTTTCAAAATGGGTCAATCGAGA
ATATCGTCAACTGTTTACTAAAAATCAGTTTCATCAAGCAATGAAACACGCCAAAGTAAACAATTTA
AGTACCGTTACTTATGAGCAAGTATTGTCTATTTTTAATAGTTATCTATTATTTAACGGGAGGAAAT
AA
>ermC
ATGAACGAGAAAAATATAAAACACAGTCAAAACTTTATTACTTCAAAACATAATATAGATAAAATA
ATGACAAATATAAGATTAAATGAACATGATAATATCTTTGAAATCGGCTCAGGAAAAGGGCATTTT
ACCCTTGAATTAGTACAGAGGTGTAATTTCGTAACTGCCATTGAAATAGACCATAAATTATGCAAAA
CTACAGAAAATAAACTTGTTGATCACGATAATTTCCAAGTTTTAAACAAGGATATATTGCAGTTTAA
ATTTCCTAAAAACCAATCCTATAAAATATTTGGTAATATACCTTATAACATAAGTACGGATATAATA
CGCAAAATTGTTTTTGATAGTATAGCTGATGAGATTTATTTAATCGTGGAATACGGGTTTGCTAAAA
GATTATTAAATACAAAACGCTCATTGGCATTATTTTTAATGGCAGAAGTTGATATTTCTATATTAAG
TATGGTTCCAAGAGAATATTTTCATCCTAAACCTAAAGTGAATAGCTCACTTATCAGATTAAATAGA
AAAAAATCAAGAATATCACACAAAGATAAACAGAAGTATAATTATTTCGTTATGAAATGGGTTAAC
AAAGAATACAAGAAAATATTTACAAAAAATCAATTTAACAATTCCTTAAAACATGCAGGAATTGAC
GATTTAAACAATATTAGCTTTGAACAATTCTTATCTCTTTTCAATAGCTATAAATTATTTAATAAGT
AA
>msrA
ATGGAACAATATACAATTAAATTTAACCAAATCAATCATAAATTGACAGATTTACGATCACTTAACA
TCGATCATCTTTATGCTTACCAATTTGAAAAAATAGCACTTATTGGGGGTAATGGTACTGGCAAAAC
CACATTACTAAATATGATTGCTCAAAAAACAAAACCAGAATCTGGAACAGTTGAAACGAATGGCGAA
ATTCAATATTTTGAACAGCTTAACATGGATGTGGAAAATGATTTTAACACGTTAGACGGTAGTTTAA
TGAGTGAACTCCATATACCTATGCATACAACCGACAGTATGAGTGGTGGTGAAAAAGCAAAATATAA
ATTAGCTAATGTCATATCAAATTATAGTCCGATATTACTTTTAGATGAACCTACAAATCACTTGGAT
AAAATTGGTAAAGATTATCTGAATAATATTTTAAAATATTACTATGGTACTTTAATTATAGTAAGT
CACGATAGAGCACTTATAGACCAAATTGCTGACACAATTTGGGATATACAAGAAGATGGCACAATAA
GAGTGTTTAAAGGTAATTACACACAGTATCAAAATCAATATGAACAAGAACAGTTAGAACAACAACG
TAAATATGAACAGTATATAAGTGAAAAACAAAGATTGTCCCAAGCCAGTAAAGCTAAACGAAATCA
AGCGCAACAAATGGCACAAGCATCATCAAAACAAAAAAATAAAAGTATAGCACCAGATCGTTTAAGT
GCATCAAAACAAAAAGGCACGGTTGAGAAGGCTGCTCAAAAACAAGCTAAGCATATTGAAAAAAGA
ATGGAACATTTGGAAGAAGTTGAAAAACCACAAAGTTATCATGAATTCAATTTTCCACAAAATAAAA
TTTATGATATCCATAATAATTATCCAATCATTGCACAAAATCTAACATTGGTTAAAGGAAGTCAAAA
ACTGCTAACACAAGTACGATTCCAAATACCATATGGCAAAAATATAGCGCTCGTAGGTGCAAATGGT
GTAGGTAAGACAACTTTACTTGAAGCTATTTACCACCAAATAGAGGGAATTGATTGTTCTCCTAAAG
TGCAAATGGCATACTATCGTCAACTTGCTTATGAAGACATGCGTGACGTTTCATTATTGCAATATTT
AATGGATGAAACGGATTCATCAGAATCATTCAGTAGAGCTATTTTAAATAACTTGGGTTTAAATGAA
GCACTTGAGCGTTCTTGTAATGTTTTGAGTGGTGGGGAAAGAACGAAATTATCGTTAGCAGTATTAT

TTTCAACGAAAGCGAATATGTTAATTTTGGATGAACCAACTAATTTTTTAGATATTAAAACATTAGA
AGCATTAGAAATGTTTATGAATAAATATCCTGGAATCATTTTGTTTACATCACATGATACAAGGTTT
GTTAAACATGTATCAGATAAAAAATGGGAATTAACAGGACAATCTATTCATGATATAACTTAA
>aac(6)-Ie+aph(2)
ATGAATATAGTTGAAAATGAAATATGTATAAGAACTTTAATAGATGATGATTTTCCTTTGATGTTA
AAATGGTTAACTGATGAAAGAGTATTAGAATTTTATGGTGGTAGAGATAAAAAATATACATTAGAA
TCATTAAAAAAACATTATACAGAGCCTTGGGAAGATGAAGTTTTTAGAGTAATTATTGAATATAAC
AATGTTCCTATTGGATATGGACAAATATATAAAATGTATGATGAGTTATATACTGATTATCATTATC
CAAAAACTGATGAGATAGTCTATGGTATGGATCAATTTATAGGAGAGCCAAATTATTGGAGTAAAG
GAATTGGTACAAGATATATTAAATTGATTTTTGAATTTTTGAAAAAAGAAAGAAATGCTAATGCAG
TTATTTTAGACCCTCATAAAAATAATCCAAGAGCAATAAGGGCATACCAAAAATCTGGTTTTAGAAT
TATTGAAGATTTGCCAGAACATGAATTACACGAGGGCAAAAAAGAAGATTGTTATTTAATGGAATA
TAGATATGATGATAATGCCACAAATGTTAAGGCAATGAAATATTTAATTGAGCATTACTTTGATAAT
TTCAAAGTAGATAGTATTGAAATAATCGGTAGTGGTTATGATAGTGTGGCATATTTAGTTAATAAT
GAATACATTTTTAAAACAAAATTTAGTACTAATAAGAAAAAAGGTTATGCAAAAGAAAAAGCAATA
TATAATTTTTTAAATACAAATTTAGAAACTAATGTAAAAATTCCTAATATTGAATATTCGTATATTA
GTGATGAATTATCTATACTAGGTTATAAAGAAATTAAAGGAACTTTTTTAACACCAGAAATTTATTC
TACTATGTCAGAAGAAGAACAAAATT
>ant(4)-Ia
ATGAGAATAGTGAATGGACCAATAATAATGACTAGAGAAGAAAGAATGAAGATTGTTCATGAAATT
AAGGAACGAATATTGGATAAATATGGGGATGATGTTAAGGCTATTGGTGTTTATGGCTCTCTTGGTC
GTCAGACTGATGGGCCCTATTCGGATATTGAGATGATGTGTGTCATGTCAACAGAGGAAGCAGAGTT
CAGCCATGAATGGACAACCGGTGAGTGGAAGGTGGAAGTGAATTTTGATAGCGAAGAGATTCTACTA
GATTATGCATCTCAGGTGGAATCAGATTGGCCGCTTACACATGGTCAATTTTTCTCTATTTTGCCGA
TTTATGATTCAGGTGGATACTTAGAGAAAGTGTATCAAACTGCTAAATCGGTAGAAGCCCAAACGTT
CCACGATGCGATTTGTGCCCTTATCGTAGAAGAGCTGTTTGAATATGCAGGCAAATGGCGTAATATT
CGTGTGCAAGGACCGACAACATTTCTACCATCCTTGACTGTACAGGTAGCAATGGCAGGTGCCATGT
TGATTGGTCTGCATCATCGCATCTGTTATACGACGAGCGCTTCGGTCTTAACTGAAGCAGTTAAGCA
ATCAGATCTTCCTTCAGGTTATGACCATCTGTGCCAGTTCGTAATGTCTGGTCAACTTTCCGACTCTG
AGAAACTTCTGGAATCGCTAGAGAATTTCTGGAATGGGATTCAGGAGTGGACAGAACGACACGGATA
TATAGTGGATGTGTCAAAACGCATACCATTTTGA
>aph(3)-IIIa
ATGAGAATATCACCGGAATTGAAAAAACTGATCGAAAAATACCGCTGCGTAAAAGATACGGAAGGA
ATGTCTCCTGCTAAGGTATATAAGCTGGTGGGAGAAAATGAAAACCTATATTTAAAAATGACGGACA
GCCGGTATAAAGGGACCACCTATGATGTGGAACGGGAAAAGGACATGATGCTATGGCTGGAAGGAAA
GCTGCCTGTTCCAAAGGTCCTGCACTTTGAACGGCATGATGGCTGGAGCAATCTGCTCATGAGTGAG
GCCGATGGCGTCCTTTGCTCGGAAGAGTATGAAGATGAACAAAGCCCTGAAAAGATTATCGAGCTGT
ATGCGGAGTGCATCAGGCTCTTTCACTCCATCGACATATCGGATTGTCCCTATACGAATAGCTTAGAC
AGCCGCTTAGCCGAATTGGATTACTTACTGAATAACGATCTGGCCGATGTGGATTGCGAAAACTGGG
AAGAAGACACTCCATTTAAAGATCCGCGCGAGCTGTATGATTTTTTAAAGACGGAAAAGCCCGAAGA
GGAACTTGTCTTTTCCCACGGCGACCTGGGAGACAGCAACATCTTTGTGAAAGATGGCAAAGTAAGT
GGCTTTATTGATCTTGGGAGAAGCGGCAGGGCGGACAAGTGGTATGACATTGCCTTCTGCGTCCGGT
CGATCAGGGAGGATATCGGGGAAGAACAGTATGTCGAGCTATTTTTTGACTTACTGGGGATCAAGCC
TGATTGGGAGAAAATAAAATATTATATTTTACTGGATGAATTGTTTTAG
